# Synthesis of a New Phenyl Chlormethine-Quinazoline Derivative, a Potential Anti-Cancer Agent, Induced Apoptosis in Hepatocellular Carcinoma Through Mediating Sirt1/Caspase 3 Signaling Pathway

**DOI:** 10.3389/fphar.2020.00911

**Published:** 2020-06-26

**Authors:** Jia-jia Lv, Wen-ting Song, Xin-min Li, Jian-mei Gao, Ze-li Yuan

**Affiliations:** ^1^ Key Laboratory of Basic Pharmacology of Ministry of Education and Joint International Research Laboratory of Ethnomedicine of Ministry of Education, Zunyi Medical University, Zunyi, China; ^2^ School of Pharmacy, Zunyi Medical University, Zunyi, China; ^3^ Guizhou International Scientific and Technological Cooperation Base for Medical Photo-Theranostics Technology and Innovative Drug Development, Zunyi Medical University, Zunyi, China

**Keywords:** hepatocellular carcinoma, phenyl chlormethine-quinazoline derivative, apoptosis, Sirt1, caspase 3

## Abstract

Quinazoline derivatives display multiple pharmacological activities and target various biological receptors. Based on the skeleton of quinazoline core, we designed and synthesized three new quinazoline-phenyl chlormethine conjugates (I–III) bearing a Schiff base (C = N) linker, and investigated their anti-tumor effects on HepG2-xenografted tumor and human cancer cell line HepG2. Among these compounds, compound II showed better inhibitory effect against HepG2 cells. In the present study, TUNEL staining, western blot, molecular docking, and siRNA were used to investigate the inhibitory mechanism of compound II towards hepatoma. Compound II inhibited HepG2-xenografted tumor growth in nude mice. Moreover, Compound II not only up-regulated Bax/Bcl-2 ratio and active-caspase 3 level, but also down-regulated Sirt1 expression and its activity, as well as PGC-1α expression. Furthermore, compound II also significantly suppressed the promotion of HepG2 cell proliferation, as evidenced by MTT assay and lactate dehydrogenase (LDH) release assay. Of note, the cytotoxicity of Compound II on HepG2 cells mainly *via* regulating Sirt1/caspase 3 signaling pathway, consisting with the results *in vivo*. Intriguingly, z-DEVD-FMK, a caspase 3 inhibitor, almost abolished the inhibitory effects of compound II. Of note, knockdown of caspase 3 by siRNA significantly reversed the inhibitory effect of compound II on HepG2. Interestingly, compound II directly bonded to Sirt1, indicating that Sirt1 might be a promising therapeutic target of compound II. In summary, our findings reveal that compound II, a new synthetical phenyl chlormethine-quinazoline derivative, contributes to the apoptosis of HepG2 cells both *in vivo* and *in vitro* through mediating Sirt1/caspase 3 singling pathway. These findings demonstrate that compound II may be a new potent agent against hepatocellular carcinoma.

## Introduction

Liver cancer, a kind of malignant lymphomas, which originates from the liver and is an invasive tumor that often occurs in the context of chronic liver disease and cirrhosis (Dimitroulis et al., 2017). Primary liver cancer or hepatocellular carcinoma (HCC) is the fifth most common cancer among men, the seventh most common cancer among women, and the leading cause of cancer-related death in the world (Prasad et al., 2020). HCC is usually diagnosed as advanced, but a lot of advanced patients cannot meet the criteria for curative treatment (Ruiz and Feray, 2015). The most common chemotherapy drugs in clinic, such as chlormethine derivatives, have serious adverse drug effects due to lacking selectivity of tumor cells (Maeda and Khatami, 2018). Moreover, unfortunately, traditional systemic chemotherapy has been also shown indistinctive efficacy and survival benefits (Li et al., 2020).

Apoptosis, termed as programmed cell death, that lead to characteristic cell changes and death, which was characterized by cell shrinkage, nuclear fragmentation, and chromatin condensation (Cikla-Suzgun and Kucukguzel, 2019). It is important to understand apoptosis in disease conditions because it not only provides insight into the pathogenesis of the disease, but also provides clues on how to treat the cancer (Abotaleb et al., 2018). Of note, tumor cell division and death are loss of balance, and cells that should die do not receive a signal to do so (Kroemer et al., 1998; Hanahan and Weinberg, 2000; Hanahan and Weinberg, 2011; Cassim et al., 2020). The problem may occur at any step in the process of apoptosis. However, apoptosis is a double-edged sword. It can be both the source of the problem and the cause of the solution, as many people are now risking new drugs for all aspects of apoptosis (Huynh et al., 2020). Therefore, apoptosis plays an important role in cancer treatment (Wong, 2011).

To overcome the limitations of nitrogen mustard drugs, researchers have chemically modified these agents with positive results. At present, the structural modification of nitrogen mustard derivatives mainly focuses on the affinity tumor heterocyclic, which are designed as a carrier to deliver the nitrogen mustard into tumor tissue (Chen et al., 2018; Chen et al., 2019). Further review of the literature reveals that among the multitudinous affinity tumor heterocyclic, quinazoline and its derivatives are often used as specific kinase inhibitors, such as tyrosine and serine kinase inhibitors (Li et al., 2013; Noolvi and Patel, 2013; Rambabu et al., 2013; Xu et al., 2013; Chen et al., 2016; Zhang et al., 2018; Zhang et al., 2019). Thus, the quinazoline pharmacophore is regarded as the ideal skeleton for developing anti-tumor agent due to two or more pharmacophores are linked through a chemical bond to a molecule to enhance the therapeutic effect of a prodrug (Domarkas et al., 2006; Milite et al., 2019; Zhang et al., 2019). Therefore, many quinazoline derivatives have been synthesized, such as those with ether, azo compound, and amine linkages, *via* the substitution of different sites in the quinazoline core to enhance the anti-cancer potency (Yin et al., 2017; Yang et al., 2018; Liu et al., 2019). Furthermore, the Schiff base group (C = N), a crucial functional group, has been revealed to markedly improve druggability because of its wide range of pharmacological effects, lower toxicity, and maximal effects (Makawana et al., 2014; Zhao et al., 2018; Sâmia et al., 2019). To date, no quinazoline-phenyl chlormethine conjugates bearing a Schiff base linker have been developed as anti-tumor agent.

In the present study, we designed and synthesized three Schiff base with a quinoline core and nitrogen mustard moieties in a single molecule to boost their anti-cancer effects, which could provide meaningful hints for discovering novel anti-cancer drug candidates.

## Materials and Methods

### Synthesis of Compounds I-III

**Figure 1:**
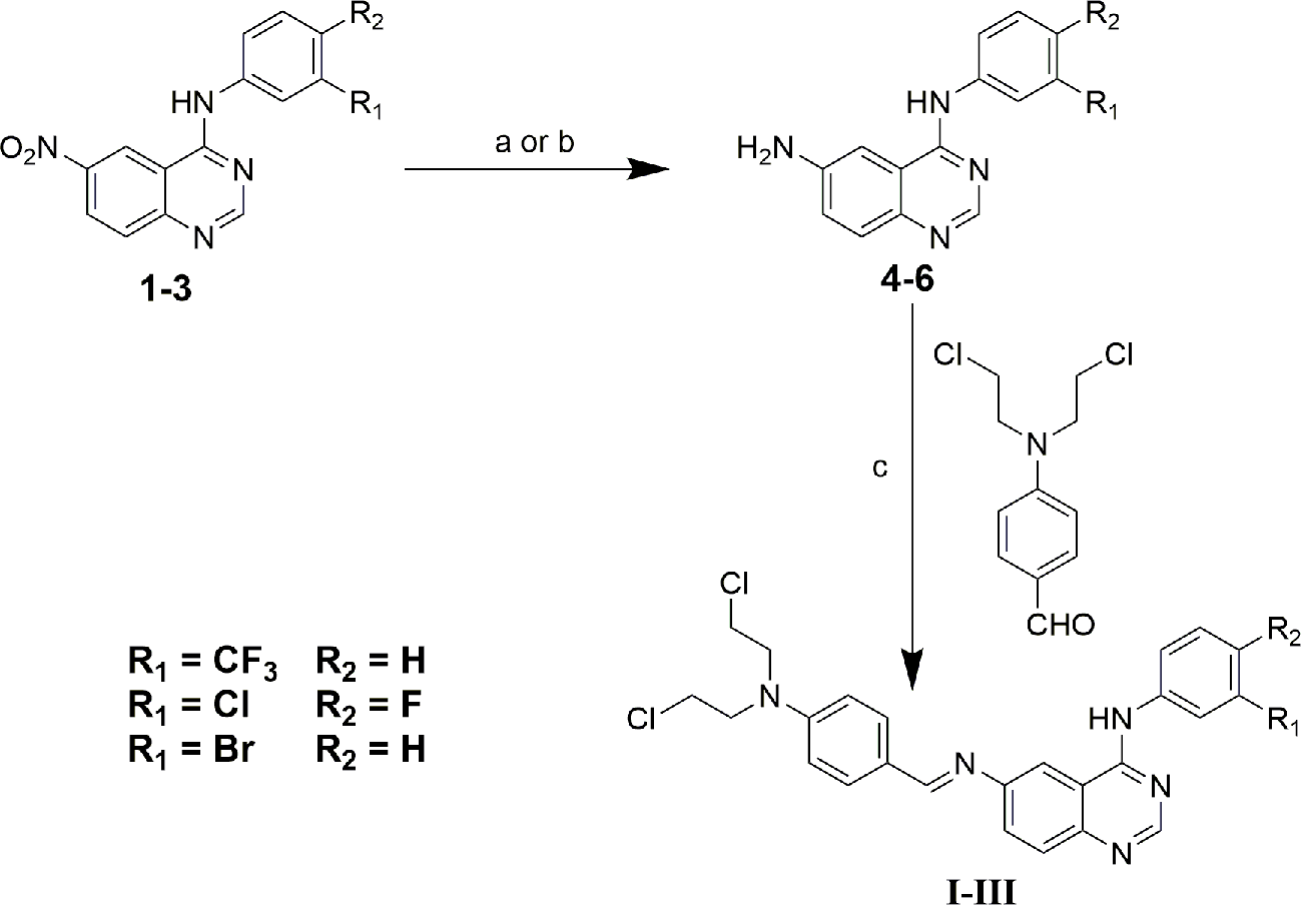
Synthetic route of compound I, II and III. (a) ethanol: tetrahydrofuran: acetic acid = 3:1:1, Pd/C/hydrazine hydrate, 50°C, 12 h; (b) ethanol: acetic acid = 5:1, Fe, 80°C, 6 h; (c) ethanol, piperidine, reflux, 48 h.

Compounds 1-3 and 4-bis (2-chloroethyl) aminobenzaldehyde were prepared as described in our previous study (Song et al., 2016). All chemical reagents and solvents were purchased from the J&K Scientific Ltd and Energy chemical company, and without further purification. ^1^H NMR and ^13^C NMR were recorded on an Agilent 400 MHz spectrometer (Agilent, Palo Alto, USA) at 25°C; High-resolution mass spectra (HRMS) were tested on a time-of-flight Micromass LCT Premier XE Spectrometer (McKinley, NJ, USA). Melting points were measured on a melting point apparatus and were uncorrected. IR spectra were recorded on a fourier transform infrared spectrometer (FT-IR1000, Varian, USA) as KBr disks over the range of 400~4000 cm^-1^. Melting points were measured on a melting point apparatus and were uncorrected. The crystal data were collected on CCD SMART APEX diffractometer at 293 K. The synthetic route of compounds I, II, and III was shown in **Figure 1**. The detailed steps and characterization were shown in supplementary information.

### HepG2-Xenografted Tumor Model

Five-week-old Male BALB/c nude mice (Vital River Laboratory Animal Technology Co., Ltd., Beijing, China) were housed in six groups in clear plastic cages and maintained on a 12 h light/dark cycle at 25 ± 1°C with water and food available *ad libitum*. All animal experiments were performed according to the experimental guidelines of the Animal Experimentation Ethics Committee of Zunyi Medical University. A number of 5 × 10^6^/ml HepG2 cells were subcutaneously implanted below the right flank of mice. Three days after the cell injection, palpable tumor size was monitored with a caliper. Xenograft tumors were allowed to grow until the average volume of the tumors reached 100 mm^3^. Tumor size (mm^3^) = length (mm) × width (mm)^2^/0.52.

### *In Vivo* Anti-Tumor Efficacy

When xenograft tumors grew to an average volume of 100 mm^3^, nude mice were randomly divided into five groups: model group, compound II low dose (1.25 mg/kg) group, compound II medium dose (2.5 mg/kg) group, compound II high dose (5 mg/kg) group, Gefitinib (10 mg/kg) group (a positive drug). Compound II (1.25, 2.5, 5 mg/kg) were injected every other day, while rats of model group were administered volume-matched normal saline. Then the body weight and tumor size were recorded at 1, 3, 5, 7, 9, 11, and 13 day. After 13th day, all the mice were executed, then the tumors were harvested, photographed, weighed, and stored at −80°C.

### Terminal-Deoxynucleoitidyl Transferase Mediated Nick End Labeling (TUNEL) Staining

The mice were treated with compound II as described above. Tumor tissues were stained according to the manufacturer’s indications.

### Western Blot

Western blot was performed as described previously (Gao et al., 2020). Briefly, the protein concentration was measured with BCA Protein Assay Kit. Samples with equal amounts of proteins were separated on 10% polyacrylamide gels, then the separated proteins were blotted onto PVDF membrane, and probed with selective antibody, respectively. The appropriate primary antibody: anti-Bax (#ab7977) (1:1,000), anti-Bcl-2 (#ab7973) (1:1,000), anti-active caspase-3 antibody (#ab49822) (1:1,000), anti-Sirt1 (#ab110304) (1:1,000), anti-PGC1 α antibody (#ab54481) (1:1,000). The optical bands were quantified by Image J software.

### Cell Culture

Human HCC tumor cell lines HepG2 were obtained from Shanghai Cell Collection (Chinese Academy of Sciences, Beijing, China) and cultured in Dulbecco’s Modified Eagles Medium supplemented with 10% fetal bovine serum,100 U/ml penicillin, and 100 μg/ml streptomycin at 37°C in a 5% CO_2_ humidified environment incubator (Thermo Scientific, USA).

### 3-(4,5-Dimethylthiazol-2-yl)-2,5-Diphenyltetrazolium Bromide (MTT) Assay

To test the growth inhibition of HepG2 by compound II, HepG2 cells were plated in 96 well plates at a density of 5 × 10^4^ cells/well and incubated at 37°C for 12 h. Then different concentrations of compound II were added into the plates. After incubation for 24 h at 37°C, the cells were washed three times with cold phosphate buffered saline, MTT at a final concentration of 5 mg/ml was added to each well following an additional culture at 37°C for 4 h. In the late incubation, culture medium was removed, and 150 μl of dimethylsulfoxide was added to dissolve formazan crystals within cells. The absorbance was measured at a wavelength of 492 nm.

### Morphological Observation

HepG2 cells in logarithmic growth phase were seeded into six-well plates at a density of 5 × 10^4^ cells/well. After incubation for 12 h, 50, 100, 200 μM compound II were added into the plates and cultured at 37°C for 24 h, cell morphology was observed under an optical microscope.

### TUNEL Staining

HepG2 cells were treated with compound II as mentioned above, and apoptosis was observed using TUNEL staining. All operations were carried out according to the manufacturer’s protocol.

### Determination of Caspase 3 Activity and Sirt 1 Activity

HepG2 cells were treated as mentioned above, the caspase 3/7 activity and Sirt 1 activity was detected by the Caspase-Glo^®^ 3/7 assay kit and Sirt 1 activity assay kit according to the manufacturer’s instructions, respectively. In brief, HepG2 cells were lysed in lysis buffer and centrifuged (12,000 × g, 5 min), then substrate working solution added into cell lysate were co-incubated in 96-well plates for 30 min at room temperature. Then the fluorescence intensity was determined with multimode reader (excitation at 490 nm wavelength and emission at 520 nm wavelength). All values of caspase 3/7 activity and Sirt 1 activity were presented as a percentage compared with the control.

### siRNA Transfection

Caspase 3-siRNA was applied to silencing endogenous expression of caspase 3 and a scrambled siRNA was used as a control. HepG2 cells were cultured at 60% confluency into 6-well plates. Caspase 3-siRNA (6 μl) and lipofectamine™ RNAiMAX transfection reagent (18 μl) were diluted into Opti-MEMI (150 μl), and thereafter mixed and cultured for 15 min at room temperature, then lipofectamine mixture was added to the caspase 3-siRNA mixture at a final concentration of 60 pM siRNA. Transfection of HepG2 cells with scrambled siRNA as a negative control. The knockdown of endogenous caspase 3 was verified using Western blot. Thereafter, the transfected cells were treated with compound II as mentioned above, and then detected the inhibition rate and LDH level.

### Molecular Docking

Molecular docking studies between compound II and Sirt1 were performed by Autodock 4.2 and Autodock Tools (ADT). The X-ray crystal structure of human Sirt1 (PDB ID: 4ZZH) was obtained from the Protein Data Bank (PDB) archives and used as target for molecular docking. The docking results were written as a pose viewer file, and the protein-ligand complex interactions were evaluated by the PyMOL molecular graphics system as described in previous study (Gao et al., 2020).

### Statistical Analysis

All the data were expressed as mean ± standard deviation (SD). Statistical comparisons were performed by one-way analysis of variance (ANOVA) for multiple groups, and *P* value < 0.05 were represented as statistically significant.

## Results

### Synthesis of Compound I~III

The synthesis of target compounds I~III was outlined in **Figure 1**. In brief, starting materials 1~3 were efficiently synthesized using microwave irradiation in our previous study (Song et al., 2016). Next, the nitro groups of 1~3 were reduced *via* disposing with Pd/C- hydrazine hydrate or Fe/acetic acid to give key intermediates 4~6. Finally, the target compounds I~III were favorably synthesized *via* condensation reaction of the Schiff base using intermediates 4~6 with 4-bis (2-chloroethyl) aminobenzaldehyde using piperidine as a catalyst. The chemical structures of all compounds were identified *via* ^1^H-NMR, ^13^C-NMR, HR-MS, and IR spectroscopy, and all experimental data were presented in the experimental and Supplementary (**Supplementary Figures 1–24**). Furthermore, compounds 5, 6, and II were further characterized *via* X-ray crystallographic analyses, the results of which were also presented in the supplementary material section (**Supplementary Figures 25–27**, **Supplementary Tables 1–4**).

### The Anti-Tumor Efficacy of Compound II

**Figure 2:**
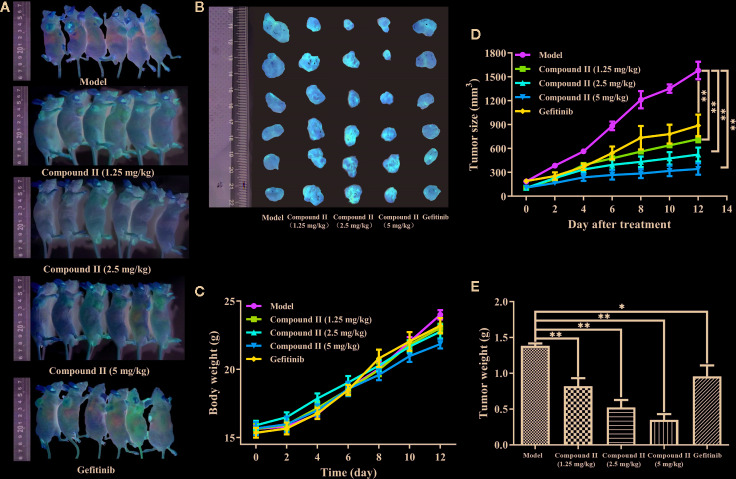
Compound II inhibited HepG2-xenografted tumor growth *in vivo*. **(A)** Photographs of HepG2 tumor-bearing mice treated with saline, gefitinib, and three doses of compound II (1.25, 2.5, and 5 mg/kg) for 12 days. **(B)** Photos of excised tumors, **(C)** the relative tumor volumes curves, **(D)** weights of excised tumors, and **(E)** body weight evolution curve. The data were presented as the mean ± SEM. ^*^ *P* < 0. 05; ^**^ *P* < 0. 05 *versus* model (n = 6).

*In vivo*, anti-tumor effect was evaluated *via* monitoring the tumor growth and detecting the apoptosis of HepG2-xenografted tumor-bearing nude mice. The results showed that compound II or Gefitinib, a positive control, significantly inhibited tumor growth (**Figures 2A, B**). Moreover, the anti-tumor effect of compound II was better than Gefitinib, as evidenced by evaluating tumor size (**Figure 2C**) and tumor weight (**Figure 2D**). Of note, the body weight of all the groups showed no differences, indicating that there were no aggressive side-effects associated with the treatments (**Figure 2E**). These findings demonstrated that compound II exhibited an obvious anti-tumor effect in a dose- dependent manner.

### Compound II Inhibited Tumor Growth in HepG2-Xenografted Tumor-Bearing Nude Mice Through Suppressing Apoptosis

**Figure 3:**
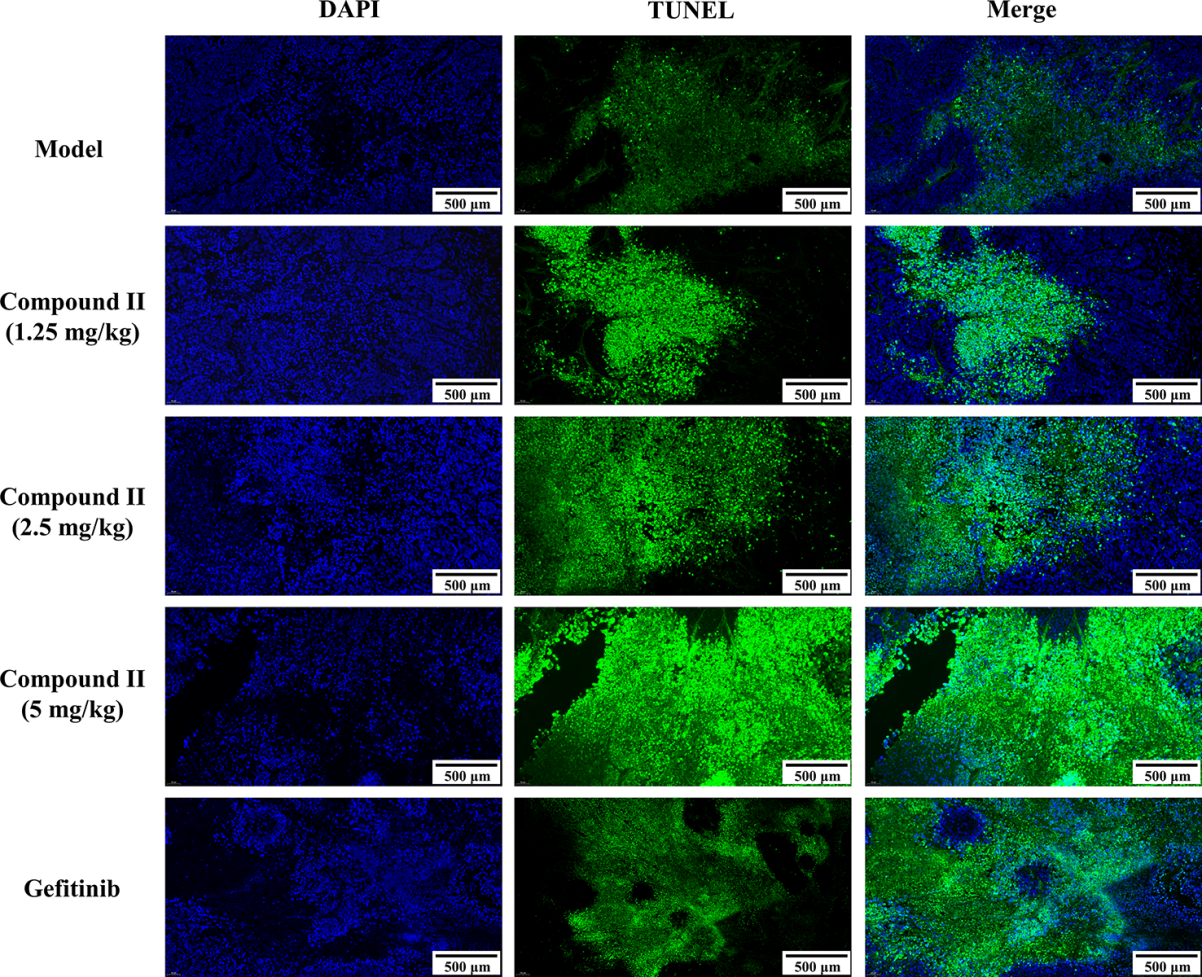
Compound II inhibited tumor growth in HepG2-xenografted tumor-bearing nude mice through suppressing apoptosis. TUNEL staining was conducted to observe the apoptosis (magnification 200 ×).

**Figure 4:**
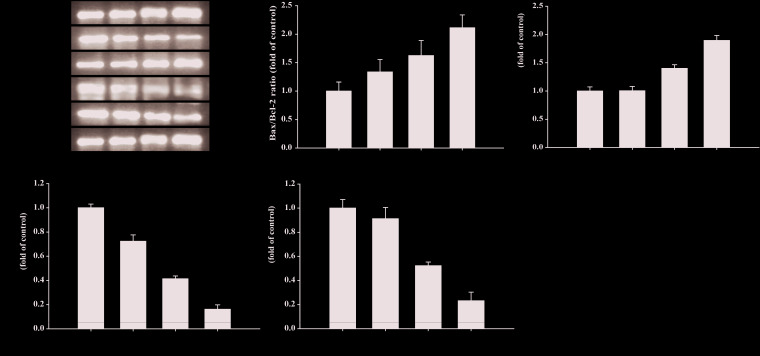
Compound II induced HepG2 (from excised tumor tissue) apoptosis through regulating Bax, Bcl-2, caspase 3, Sirt1, and PGC-1 α. **(A)** Representative Western blot were shown for Bax, Bcl-2, caspase 3, Sirt1, and PGC-1 α. **(B)** Quantitation of phosphorylation of Bax/Bcl-2. **(C)** Quantitation of active-caspase 3 protein. **(D)** Quantitation of Sirt1 protein. **(E)** Quantitation of PGC-1 α protein. Caspase 3-specific inhibitor significantly abolished the inhibitory effect of compound II (n = 3). The data were presented as the mean ± SEM. ^*^ *P* < 0. 05; ^**^ *P* < 0. 05 *versus* model.

The results demonstrated that compound II (5 mg/kg) or Gefitinib significantly induced apoptosis than that of model group, as evidenced by TUNEL staining (**Figure 3**). Furthermore, compound II (5 mg/kg) not only up-regulated the ratio of Bax/Bcl-2 (**Figures 4A, B**), but also increased the level of active-caspase 3 (**Figure 4C**). Moreover, compound II (5 mg/kg) markedly decreased the expressions of Sirt1 (**Figure 4D**) and PGC-1α (**Figure 4E**).

### The Inhibitory Effect of Compound II on HepG2 Cells *In Vitro*

**Figure 5:**
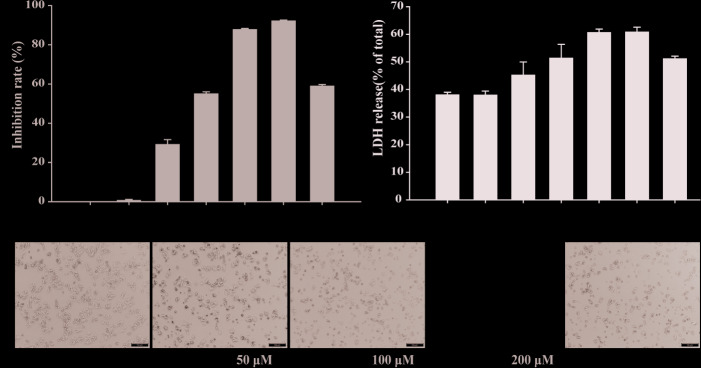
The effects of compound II on inhibition rate and morphological characteristics on HepG2 cells. HepG2 cells treated with various concentrations of compound II for 24 h. **(A)** cell viability. **(B)** LDH release. Data were presented as mean ± SD of three independent experiments. ^*^ *P* < 0.05, ^**^ *P* < 0.01 *versus* untreated control cells. **(C)** Morphological changes of HepG2 cells observed under phase-contrast microscopy after treating with and without compound II for 24 h (magnification 200 ×).

The results showed that compound II inhibited cell growth of HepG2 cells in a concentration-dependent manner (**Figure 5A**), In parallel, LDH release was significantly increased in the compound II -treated cells than that of the control group (**Figure 5B**), but compound I and compound III did not affect the LDH level (**Supplementary Figure 28**). The effect of compound II was also confirmed by morphologic observations (**Figure 5C**). These results indicated that compound II exhibited inhibitory effects on HepG2 cells, consisting with the results *in vivo*.

### Compound II Induced Apoptosis in HepG2 Cells Through Sirt1/Caspase 3 Signaling Pathway

**Figure 6:**
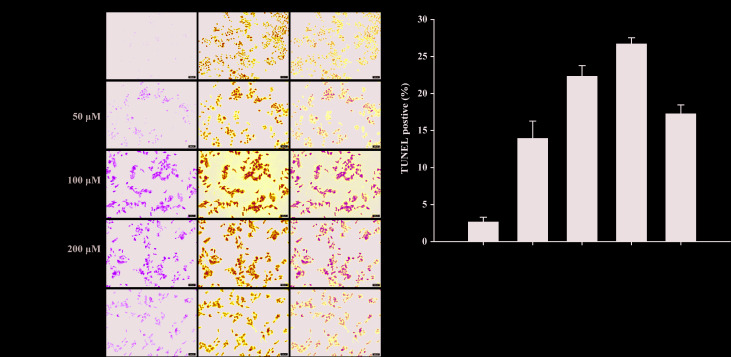
Apoptosis detection in HepG2 cells treated with compound II. **(A)** Representative photographs of TUNEL staining in different cases. Cells were detected and analyzed with TUNEL (green) and counterstained with DAPI (blue). **(B)** Quantitative analysis of TUNEL positive cells. The percentage of TUNEL positive cells was expressed as percent DAPI stained cells. Data were presented as mean ± SD of three independent experiments. ^**^ *P* < 0.01 *versus* untreated control cells.

**Figure 7:**
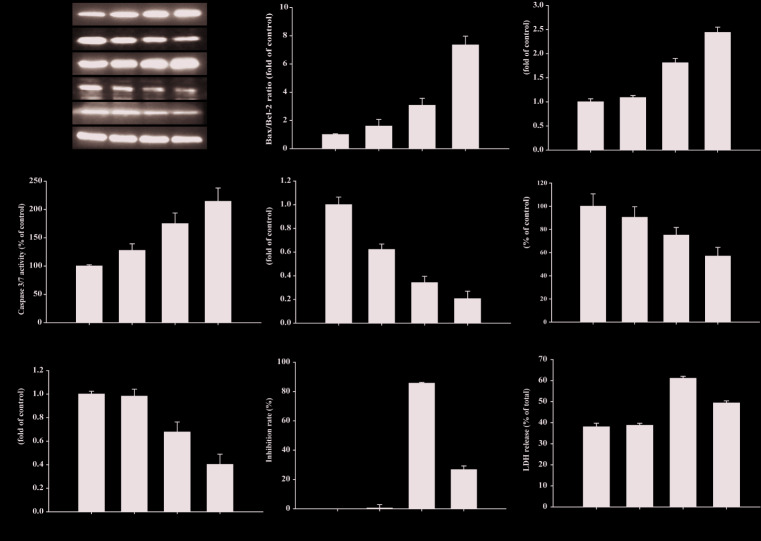
Compound II induced HepG2 apoptosis through regulating Bax, Bcl-2, caspase 3, Sirt1, and PGC-1 α. **(A)** Representative Western blot were shown for Bax, Bcl-2, caspase 3, Sirt1, and PGC-1 α. **(B)** Quantitation of phosphorylation of Bax/Bcl-2. **(C)** Quantitation of active-caspase 3 protein. **(D)** Caspase 3/7 activity. **(E)** Quantitation of Sirt1 protein. **(F)** Sirt 1 activity. **(G)** Quantitation of PGC-1 α protein. Caspase 3-specific inhibitor significantly abolished the inhibitory effect of compound II. **(H)** Inhibition rate was determined using MTT assay. **(I)** LDH release was determined using an LDH release assay. Data were presented as mean ± SD of three independent experiments. ^*^ *P* < 0.05, ^**^ *P* < 0.01 *versus* untreated control cells. ^#^ *P* < 0.05, ^##^ *P* < 0.01 *versus* compound II.

The results further showed that compound II induced the apoptosis in HepG2 cells in a dose-dependent manner than that of model group, as evidenced by TUNEL staining, which was consistent with the results *in vivo* (**Figure 6**). Furthermore, compound II or Gefitinib not only up-regulated the ratio of Bax/Bcl-2 (**Figures 7A, B**), but also increased the level of active-caspase 3 (**Figures 7A, C**), as well as caspase 3 activity (**Figure 7D**). Moreover, compound II markedly decreased the expressions of Sirt1 (**Figures 7A, E**) and PGC-1α (**Figures 7A, G**), as well as Sirt 1 activity (**Figure 7F**), which were in line with the findings *in vivo*. Intriguingly, z-DEVD-FMK, a caspase 3-specific inhibitor, significantly abolished the inhibitory effect of compound II, as evidenced by MTT assay and LDH release, respectively (**Figures 7H, I**). Furthermore, siRNA was applied to further verified that compound II activated caspase 3 to induced HepG2 apoptosis. The results showed that caspase 3 siRNA treated cells exhibited lower level of caspase 3 than that of transfected control cells and scrambled siRNA transfected cells (**Supplementary Figure 29A**). Of note, the inhibition rate and LDH level of caspase 3 siRNA transfected cells were markedly decreased than that of scrambled siRNA transfected cells (**Supplementary Figures 29B, C**). These findings suggested that further confirming that Sirt1/caspase 3 signaling pathway was involved in the inhibitory effects of compound II on HepG2 cells.

### Compound II Directly Bound to Sirt1

**Figure 8:**
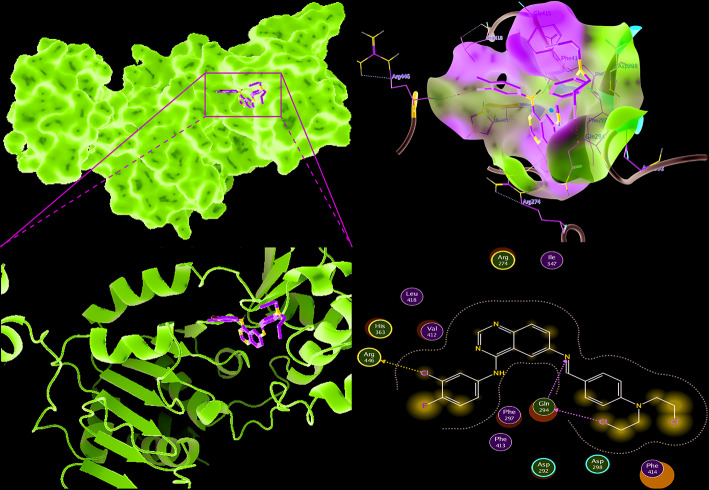
The binding energy has been surfaced and key residues of compound II with Sirt1 were displayed by molecular docking. **(A)** The surface of substrate binding. **(B)** The ribbon of substrate binding sites. **(C)** The pocket of substrate binding. **(D)** Amino acid residues. Green stick indicated compound II, purple surface or ribbon indicated Sirt1.

The binding affinity of compound II and Sirt1 was detected by molecular docking analysis. The results demonstrated that the binding energy of compound II with Sirt1 was −8.23 kcal/mol, and the suppositive binding modes and interaction within the amino acid pocket between compound II and Sirt1 mainly including Gln 294, Phe 297, Asp292, Val 412, Arg 274, Leu 418 (**Figure 8**). These findings indicated that compound II might be directly bind to Sirt1.

## Discussion

**Figure 9:**
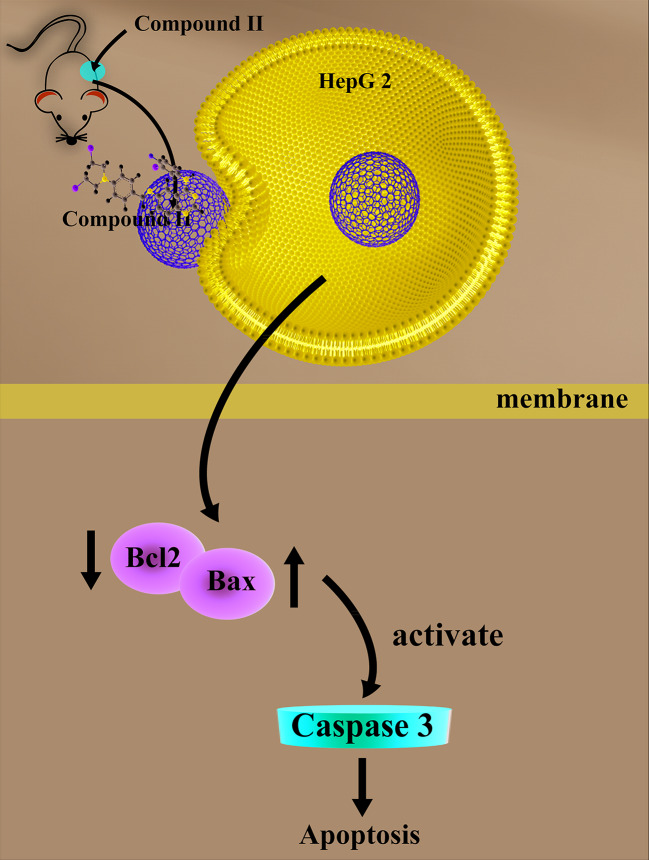
The schematic presentation elucidating proposed mechanisms that compound II induces apoptosis both *in vivo* and *in vitro*, at least partly, through activating caspase 3 *via* promoting Sirt1/PGC-1α signaling pathway.

The current study demonstrated for the first time that: (1) compound II exerted inhibitory effects on HCC both *in vivo* and *in vitro*; (2) The inhibitory effects of compound II on HCC through suppressing apoptosis; (3) Most intriguingly, compound II not only inactivated caspase 3, but also regulated Sirt1/PGC-1α pathway (**Figure 9**).

In the present study, we developed three new quinazoline-phenyl chlormethine conjugates (I–III) bearing a Schiff base (C = N) linker. We found that compound II significantly inhibited HCC in HepG2-xenografted tumor and HepG2 proliferation both *in vivo* and *in vitro*, suggesting that compound II exhibited potent inhibitory effect on HCC. However, its underlying mechanisms remains still unclear.

Apoptosis, termed as programmed cell death, that leads to characteristic cell changes and death, including cell shrinkage, nuclear fragmentation, chromatin condensation (Elmore, 2007). Accumulating evidence indicates that excessive apoptosis causes aging or neurodegenerative diseases, whereas an inadequate apoptosis results in uncontrollable cell proliferation, such as HCC (Guicciardi and Gores, 2005). The findings in the present study showed that compound II not only inhibited apoptosis in HepG2-xenografted tumor model *in vivo*, but also suppressed cell proliferation *via* inducing apoptosis in HepG2 *in vitro*, as evidenced by TUNEL staining. Whereas, its detailed mechanism was needed explored in depth.

Bcl-2, encoded in humans by the Bcl-2 gene, is a member of the Bcl-2 family of regulator proteins that regulate apoptosis, such as pro-apoptotic protein Bax and anti-apoptotic protein Bcl-2 (Tsujimoto, 1998; Yip and Reed, 2008). Increased Bax/Bcl-2 ratio leads to activation of caspase 3, which plays a vital role in the execution-phase of cell apoptosis (Porter and Janicke, 1999). Notably, compound II enhanced Bax/Bcl-2 ratio and the level of active-caspase 3, indicating that compound II inhibited HCC, at least partly, through mediating caspase-3-dependent apoptosis, as evidenced by Western blot, caspase 3 inhibitor, and caspase 3 siRNA. Most importantly, Sirt1 belongs to the nicotinamide adenine dinucleotide (NAD)-dependent histone deacetylases (Rahman and Islam, 2011). Sirt1 plays an important role in multiple signaling pathways, including cell proliferation, apoptosis, and so on (Liu et al., 2017). Notably, mounting reports suggest that Sirt1 expression is increased in HCC, as well as PGC-1α, which are promoted deacetylation by Sirt1 (Moon, 2011; Man et al., 2012). In the present study, we found that, compound II downregulated the expressions of Sirt1 and PGC-1α, suggesting that the inhibitory effect of compound II on HCC may be related to regulate Sirt1/PGC-1α pathway. Intriguingly, compound II directly bound to Sirt1, indicating that Sirt1 might be the potential target of compound II. Of note, since PGC-1αis the vital protein in the control of mitochondrial biogenesis, compound II may then eventually affect HCC cell mitochondrial functions and bioenergetics, and that will be further elucidated in our future studies (Cassim et al., 2018a; Cassim et al., 2018b).

It should be noted that this study has revealed the inhibitory effect of compound II on hepatoma and its preliminary mechanisms. However, whether compound II can also display the same inhibitory effect on hepatoma in immunocompetent mice, and its unequivocal target still need to be in-depth explored. In fact, these problems could be solved by Hepa1-6 or HepG2 caspase 3 null cells -xenografted tumor model with B16 immunocompetent mice, and well differentiated human Huh7 or HepaRG cells in our next story.

In conclusion, the present study reveals that compound II induces apoptosis through mediating Sirt1/caspase 3 signaling pathway. These findings demonstrate that compound II may be a promising potent agent against HCC.

## Data Availability Statement

The raw data supporting the conclusions of this article will be made available by the authors, without undue reservation, to any qualified researcher.

## Author Contributions

Z-LY and J-MG designed the experimental protocols. J-JL carried out all the studies with help from W-TS and X-ML. J-JL wrote the manuscript with help from Z-LY and J-MG.

## Funding

Thanks to funding from the Natural Science Foundation of China (Grant No. 81660575, 81360471), Innovative Group Project of Guizhou Province of Education (KY[2018]024), Academic Seedling Program of Zunyi Medical University ([2017]5733-042), and the Doctor Foundation of Zunyi Medical University (F-862), the ministry of education “ChunHui Plan” research projects (Z2017003), and Science and Technology Plan of Zunyi ([2018]13), Talents of Guizhou Science and Technology Cooperation Platform [2020]4104.

## Conflict of Interest

The authors declare that the research was conducted in the absence of any commercial or financial relationships that could be construed as a potential conflict of interest.
