# Supplementary material for: Synthesis of a New Phenyl Chlormethine-Quinazoline Derivative, a Potential Anti-Cancer Agent, Induced Apoptosis in Hepatocellular Carcinoma Through Mediating Sirt1/Caspase 3 Signaling Pathway
Source: Front Pharmacol. 2020 Jun 26;11:911. doi: 10.3389/fphar.2020.00911 (PMC7332554; doi:10.3389/fphar.2020.00911)
Supplement: Supplementary file 1 [file DataSheet_1.docx]

Supplementary Material

# Supplementary Data

The compounds 4 and 5 were prepared by using Pd/C/hydrazine hydrate as catalyst: to a solution of the compound 1 (8.25 mmol) in tetrahydrofuran (10 mL), ethanol (30 mL) and acetic acid (10 mL) mixture solutions were added Pd/C (10%, 0.1 g) and hydrazine hydrate (3 mL), and the resulting solutions was heated up to 50 ℃ for 12 h. After finish of the reaction, the Pd/C can be removed by filter. The filtrate was condensed to a quarter of its volume, and the compound 4 was obtained by recrystallization using dimethyl sulfoxide. Yellow, yield 67%, M.p. 199～201 ℃. ^1^H NMR (DMSO-*d*_6_, 400 MHz), *δ* (ppm): 9.62 (s, 1H, NH); 8.40 (s, 1H, CH-N), 8.34 (s, 1H, Ar-H), 8.22～8.24 (d, 1H, *J*=8.3 Hz, Ar-H), 7.85～7.60 (dd, 2H, *J*=12.6, 5.9 Hz, Ar-H), 7.37～7.39 (t, 2H, *J*=5.1 Hz, Ar-H), 7.26～7.29 (dd, 1H, *J*=8.9, 2.3 Hz, Ar-H), 5.66 (s, 2H, NH_2_); ^13^C NMR (DMSO-*d*_6_, 100 MHz), *δ* (ppm): 168.47, 155.75, 149.53, 147.53, 140.93, 129.53, 129.35, 128.83, 123.97, 118.83, 117.31, 116.78, 100.82, 20.54; HRMS (ESI-MS): Exact mass calcd for C_15_H_12_F_3_N_4_ [M+H]^+^ 305.10086, found 305.10082.

The compound 5 was synthesized by using the same method as that for 4. Green crystal, yield 74%, M.p. 249～251 ℃. ^1^H NMR (DMSO-*d*_6_, 400 MHz), *δ* (ppm): 9.49 (s, 1H, NH), 8.95 (s, 2H, CH-N and Ar-H), 8.35 (s, 1H, Ar-H), 8.19～8.21 (dd, 2H, *J*=6.8, 2.4 Hz, Ar-H), 7.80～7.84 (m, 1H, Ar-H), 7.53～7.55 (d, 1H, *J*=8.9 Hz, Ar-H), 7.38～7.42 (t, 1H, *J*=8.9 Hz, Ar-H), 7.32～7.33 (d, 1H, *J*=2.2 Hz, Ar-H), 7.24～7.27 (m, 1H, Ar-H), 5.64 (s, 2H, NH_2_); ^13^C NMR (DMSO-*d*_6_, 100 MHz), *δ* (ppm): 168.80, 155.79, 151.73, 149.62, 147.53, 128.80, 122.88, 121.88, 121.81, 118.85, 116.65, 116.44, 100.84, 20.57; HRMS (ESI-MS): Exact mass calcd for C_14_H_11_ClFN_4_ [M+H]^+^ 289.06508, found 289.06540.

The compound 6 was obtained by using Fe/acetic acid as catalyst: to a mixture of the compound 3 (14.94 g, 43.3 mmol) and Fe (12.0 g, 210 mmol) were suspend in ethanol (75 mL) and acetic acid (15 mL), and the resulting solution was kept under 80 ℃ for 6 h. After reaction is completed, the reaction is naturally cooled to room temperature, diluted with double-distilled water, and filtered through decompressor. The resulting yellow solid washed with water and the result solid was further dissolved in ethanol (8 mL), tetrahydrofuran (38 mL), and ethyl acetate (105 mL). After dissolution is finished, the undissolved substance can be dislodged by filter. Then, the filtrate washed with 25% K_2_CO_3_ and saturated NaCl until neutrality, and the organic phase was dried over anhydrous Na_2_SO_4_ and evaporated to give crude 6. A yellow crystal 6 was obtained by recrystallization using dimethyl sulfoxide. Yield 43%, M.p. 196～198 ℃. ^1^H NMR (DMSO-*d*_6_, 400 MHz), δ (ppm): 9.46 (s, 1H, NH), 8.39 (s, 1H, CH-N), 8.24～8.25 (t, 1H, *J*=1.8 Hz, Ar-H), 7.88～7.90 (d, 1H, *J*=1.8 Hz, Ar-H), 7.54～7.57 (dd, 1H, *J*=8.9, 1.6 Hz, Ar-H), 7.31～7.35 (m, 1H, Ar-H), 7.22-7.26 (m, 2H, Ar-H), 5.64 (s, 2H, NH_2_); ^13^C NMR (DMSO-*d*_6_, 100 MHz), δ (ppm): 155.69, 147.44, 142.71, 141.77, 130.30, 128.78, 125.12, 120.05, 116.73, 100.83; HRMS (ESI-MS): Exact mass calcd for C_14_H_12_BrN_4_ [M+H]^+^ 315.02399, found 315.02417.

To a solution of 4-bis(2-chloroethyl)aminobenzaldehyde (0.25 g, 1 mmol) and compound 4 (1 mmol) ethanol (30 mL) was added 7 drops piperidine , and the mixture solution was refluxed for 48 h. Next, the reaction solution was reduced to about 8 mL, purified by column flash chromatography on silica gel (petroleum ether: ethyl acetate =1:5, *v*:*v*). Then the title compound I was obtained by crystallization from ethanol to provide I as yellow solid. Yield: 65%, M.p. 197～199℃. ^1^H NMR (DMSO-*d*_6_, 400 MHz), *δ* (ppm): 9.99 (s, 1H, NH), 8.63～8.65 (d, 2H, *J*=6.0 Hz, CH=N), 8.37-8.39 (d, 2H, *J*=6.0 Hz, Ar-H), 8.32～8.34 (d, 1H, *J*=8.3 Hz, Ar-H), 7.84～7.87 (d, 4H, *J*=9.4 Hz, Ar-H), 7.62～7.66 (t, H, *J*=8.0 Hz, Ar-H), 7.45～7.47 (d, 1H, *J*=7.8 Hz, Ar-H), 6.92～6.94 (d, 2H, *J*=9.0 Hz, r-H), 3.81～3.86 (m, 8H, 4×CH_2_); ^13^C NMR (DMSO-*d*_6_, 100 MHz), *δ* (ppm): 161.05, 157.39, 150.24, 147.87, 140.28, 129.63, 127.66, 124.73, 117.73, 115.69, 113.57, 111.75, 51.83, 40.97; HRMS(ESI-MS): Exact mass calcd for C_26_H_23_Cl_2_F_3_N_5_ [M+H]^+^ 532.1277 found 532.1212. FT-IR (KBr,4000～400 cm^-1^) δ: 3474, 3416, 1619, 1529, 1401, 1327,1252, 1164, 617.

The similar method was used for preparation II and III. Compound II, red, yield 52%, M.p. 206～208℃. ^1^H NMR(DMSO-d_6_, 400 MHz), *δ*(ppm): 9.84(s, 1H, NH), 8.60(s, 2H, CH=N), 8.30(s, 1H, Ar-H), 8.25～8.27 (m, 1H, Ar-H), 7.88～7.91(m, 1H, Ar-H), 7.80～7.84(m, 4H, Ar-H), 7.41～7.46(t, 1H, *J*=9.1 Hz, Ar-H), 6.90～6.92(d, 1H, *J*=8.7 Hz, Ar-H), 3.80～3.84(m, 8H, 4×CH_2_); ^13^C NMR(DMSO-d_6_, 100 MHz), *δ*(ppm): 160.94, 157.29, 154.43, 153.19, 152.01, 150.12, 149.43, 147.79, 136.66, 136.63, 130.82, 124.73, 123.26, 122.14, 122.07, 118.88, 118.70, 116.65, 116.43, 115.57, 113.55, 111.73, 56.04, 51.84, 40.96, 18.57; HRMS(ESI-MS): Exact mass calcd for C_25_H_22_Cl_3_FN_5_ [M+H]^+^ 516.0909, found 516.0890. FT-IR(KBr, 4000～400 cm^-1^) δ: 3474, 3416, 1619, 1529, 1401, 1327, 1252, 1164, 617.

Compound III, Orange, yield 61%, M.p. 189～191℃. ^1^H NMR (DMSO-*d*_6_, 400 MHz), *δ* (ppm): 9.84 (s, 1H, NH), 8.62 (s, 2H, CH=N), 8.28～8.34 (d, 2H, *J*=24.6 Hz, Ar-H), 7.95～7.97 (d, 1H, *J*=8.1 Hz, Ar-H), 7.82～7.85 (m, 4H, Ar-H), 7.29～7.38 (m, 2H, Ar-H), 6.91～9.63 (d, 1H, *J*=8.8 Hz, Ar-H), 3.80～3.84 (m, 8H, 4×CH_2_); ^13^C NMR (DMSO-*d*_6_, 100 MHz), *δ* (ppm): 161.49, 157.75, 150.62, 148.26, 141.50, 131.28, 126.30, 124.36, 120.90, 116.11, 113.94, 112.16, 56.46, 52.24, 40.01, 19.00; HRMS (ESI-MS): Exact mass calcd for C_25_H_23_BrCl_2_N_5_ [M+H]^+^ 542.0508, found 542.0488 FT-IR (KBr, 4000～400 cm^-1^) δ: 3417, 3230, 3177, 1610, 1522, 1399, 1252, 1166, 1090, 992, 839.

# Supplementary Figures and Tables

## **Supplementary Figures**

### NMR spectroscopies of 4-5

**Supplementary Figure 1.** The ^1^H NMR spectroscopy of **4** in DMSO-d_6_

**Supplementary Figure 2.** The ^13^C NMR spectroscopy of **4** in DMSO-d_6_

**Supplementary Figure 3.** The ^1^H NMR spectroscopy of **5** in DMSO-d_6_

**Supplementary Figure 4.** The ^13^C NMR spectroscopy of **5** in DMSO-d_6_


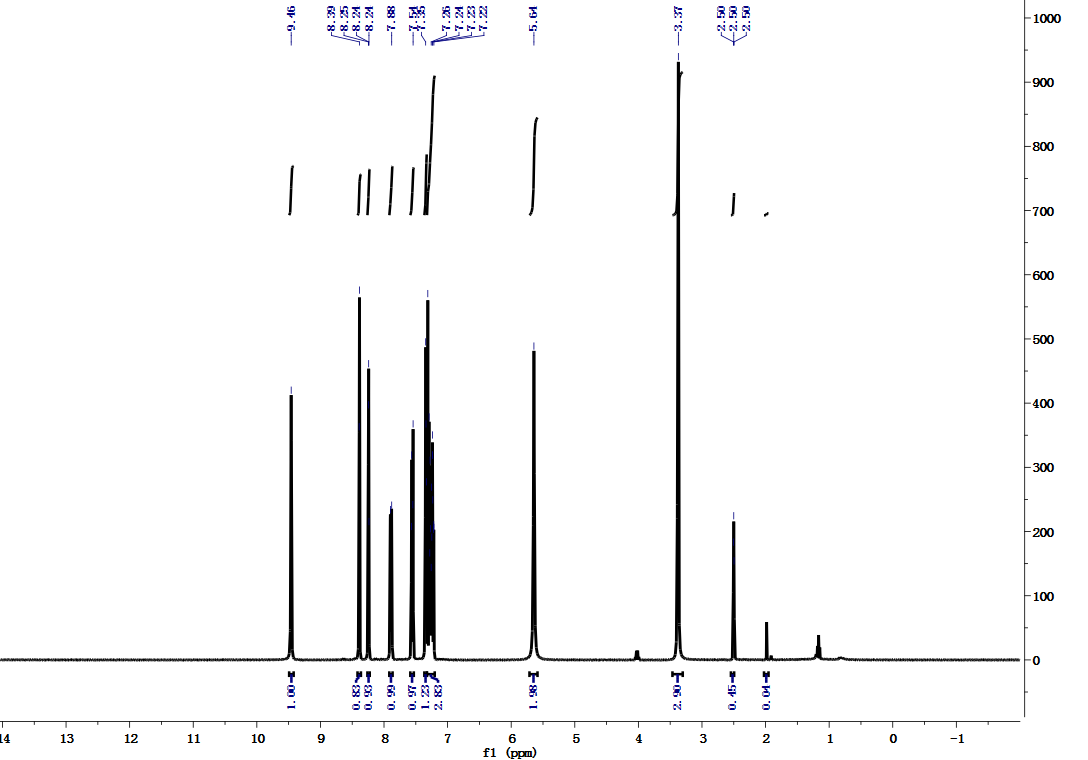

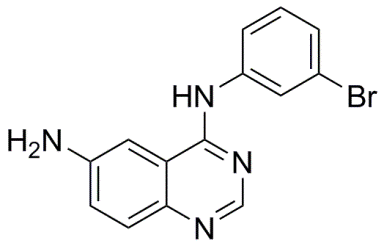


**Supplementary Figure 5.** The ^1^H NMR spectroscopy of **6** in DMSO-d_6_


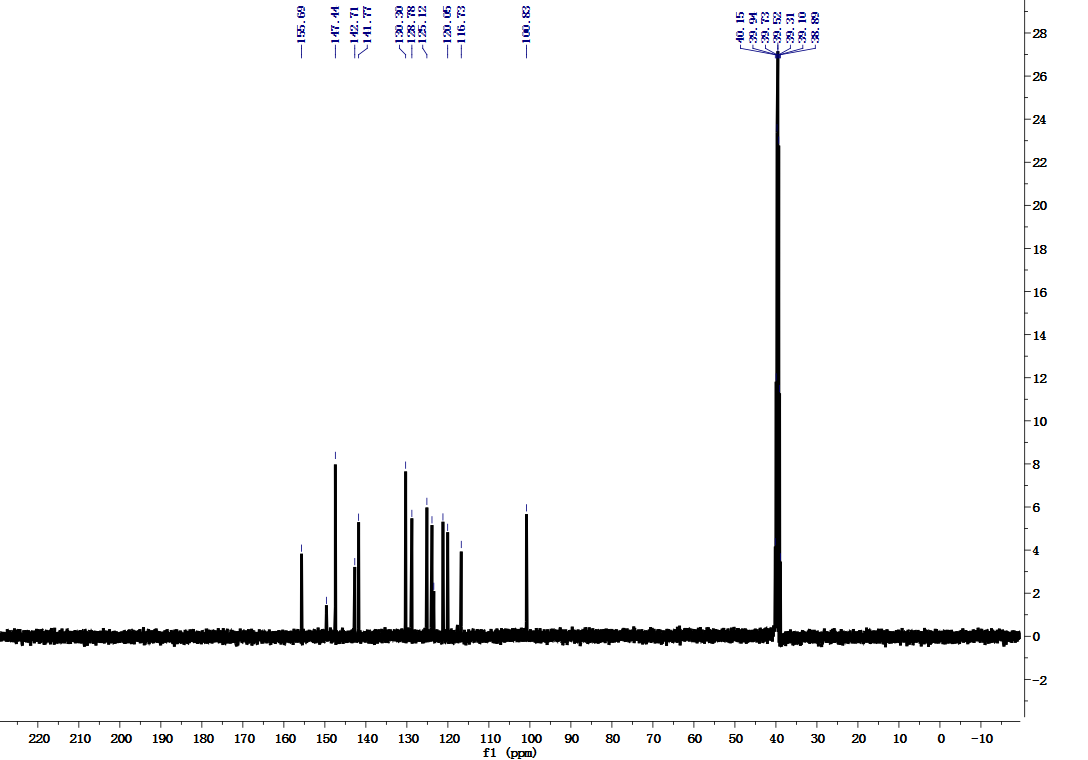

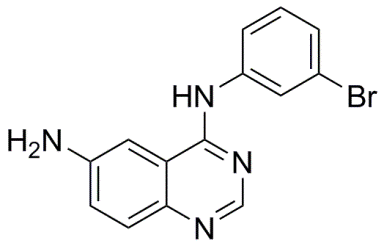


**Supplementary Figure 6.** The ^13^C NMR spectroscopy of **6** in DMSO-d_6_

### NMR spectroscopies of I-III


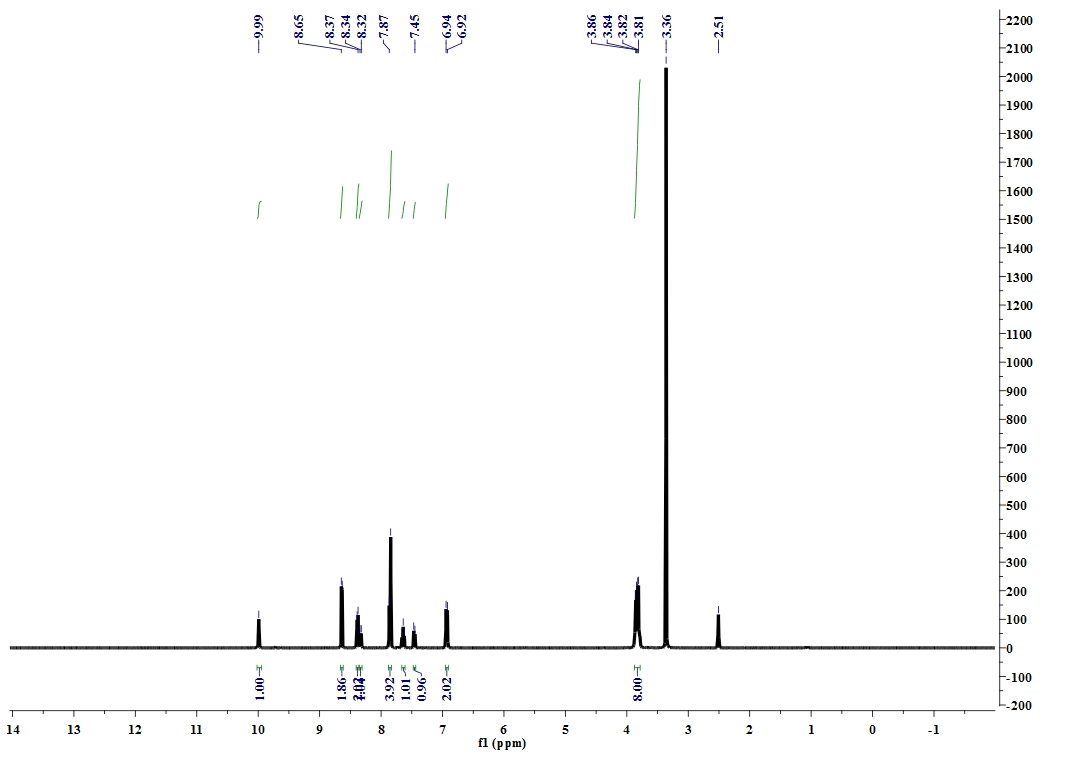

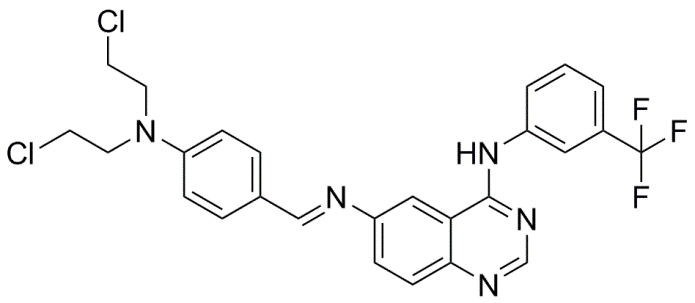


**Supplementary Figure 7.** The ^1^H NMR spectroscopy of **I** in DMSO-d_6_


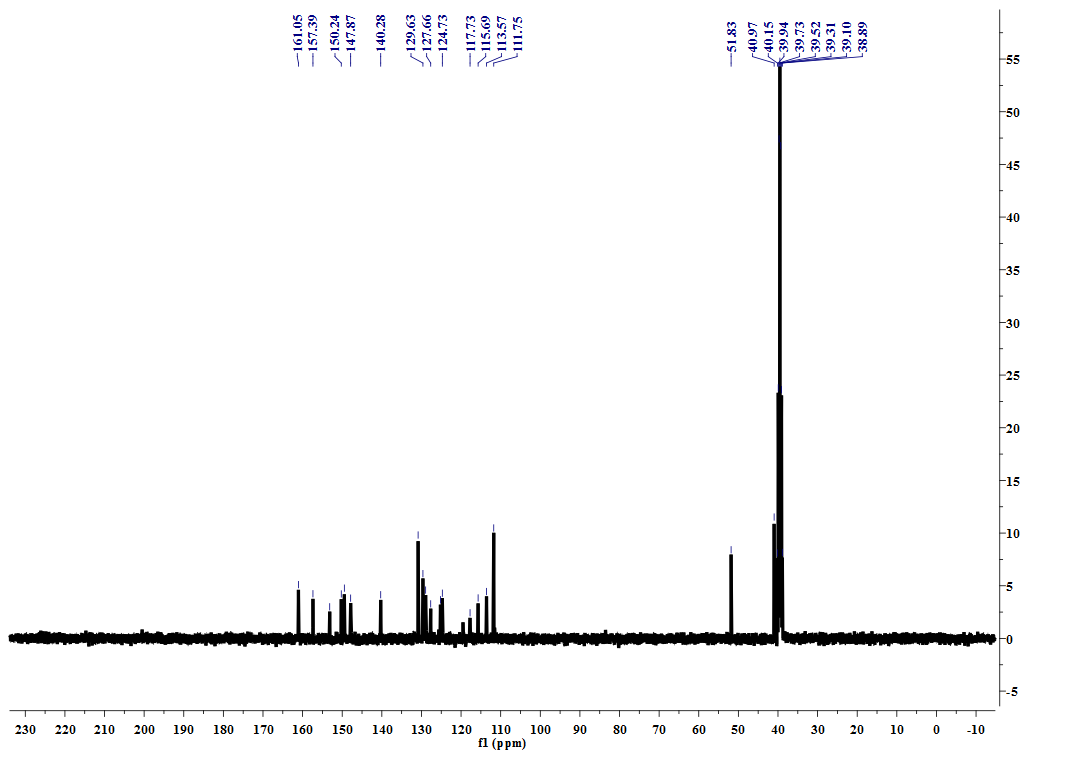

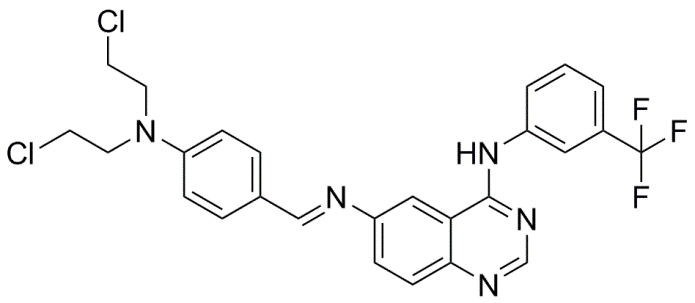


**Supplementary Figure 8.** The ^13^C NMR spectroscopy of **I** in DMSO-d_6_


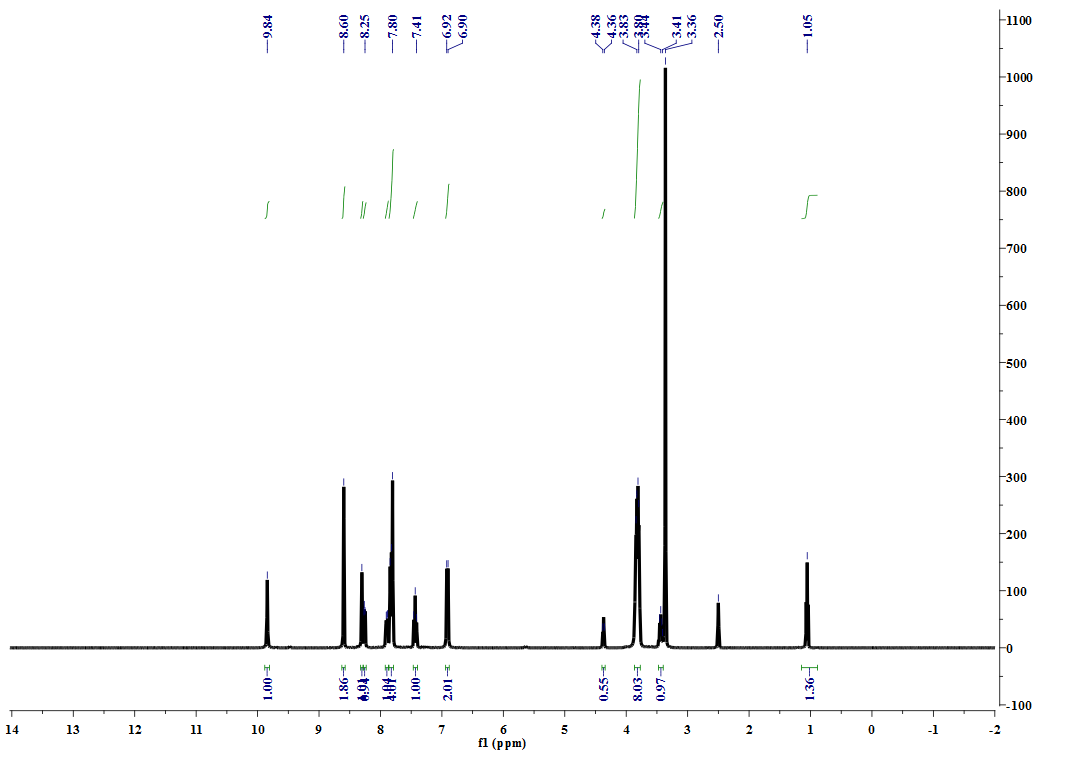

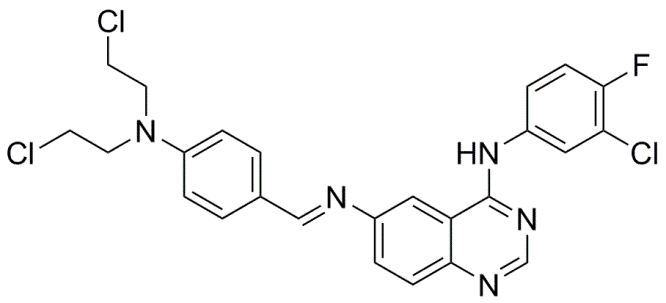


**Supplementary Figure 9.** The ^1^H NMR spectroscopy of **II** in DMSO-d_6_


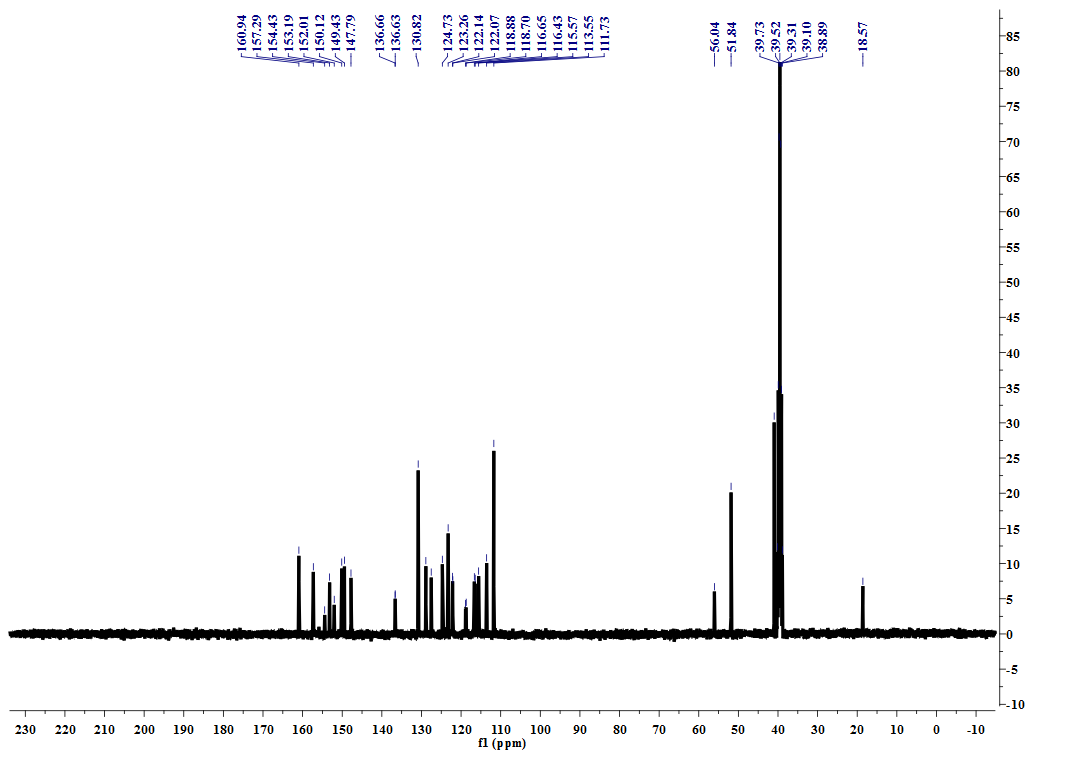

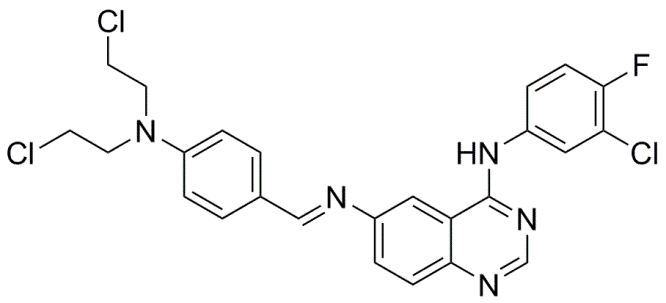


**Supplementary Figure 10.** The ^13^C NMR spectroscopy of **II** in DMSO-d_6_


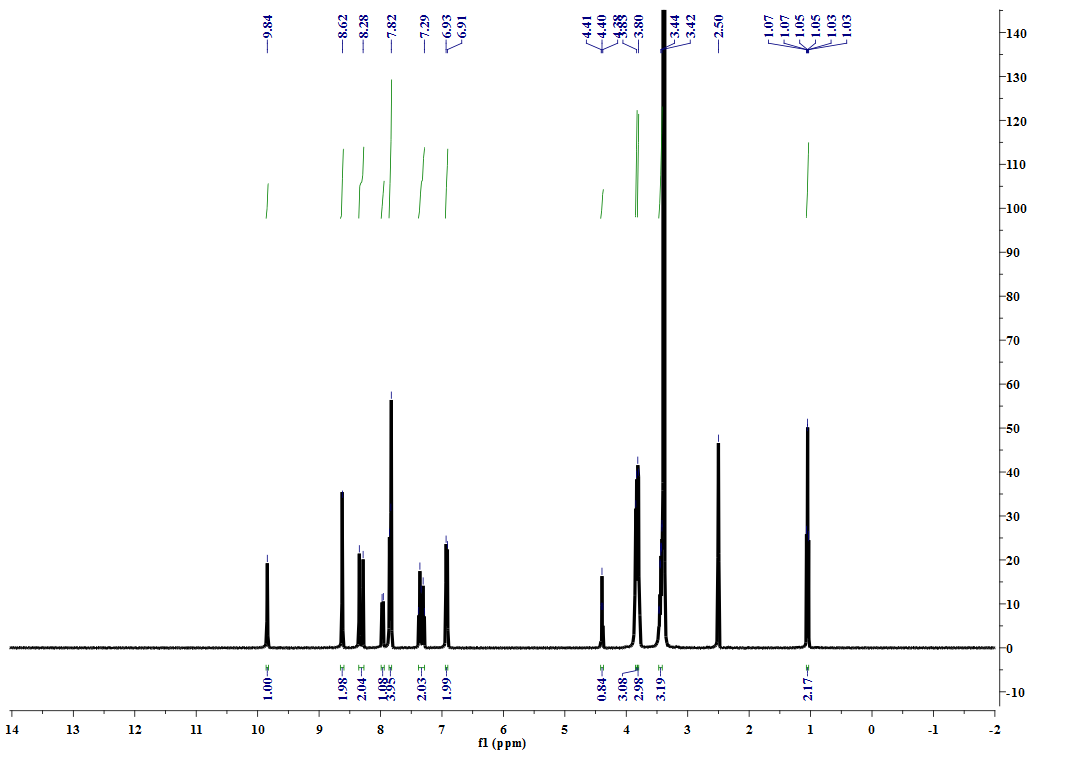

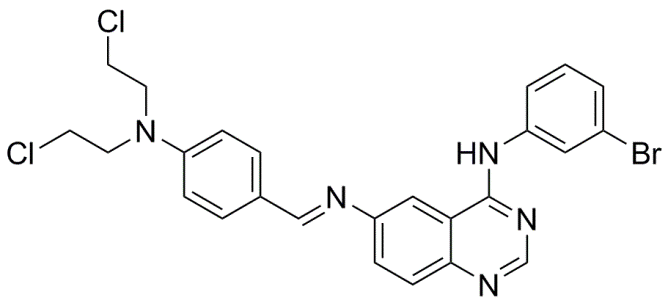


**Supplementary Figure 11.** The ^1^H NMR spectroscopy of **III** in DMSO-d_6_


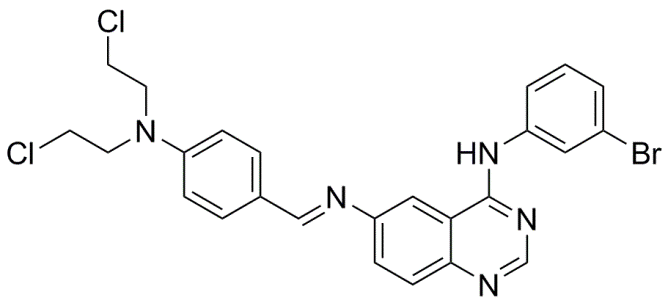

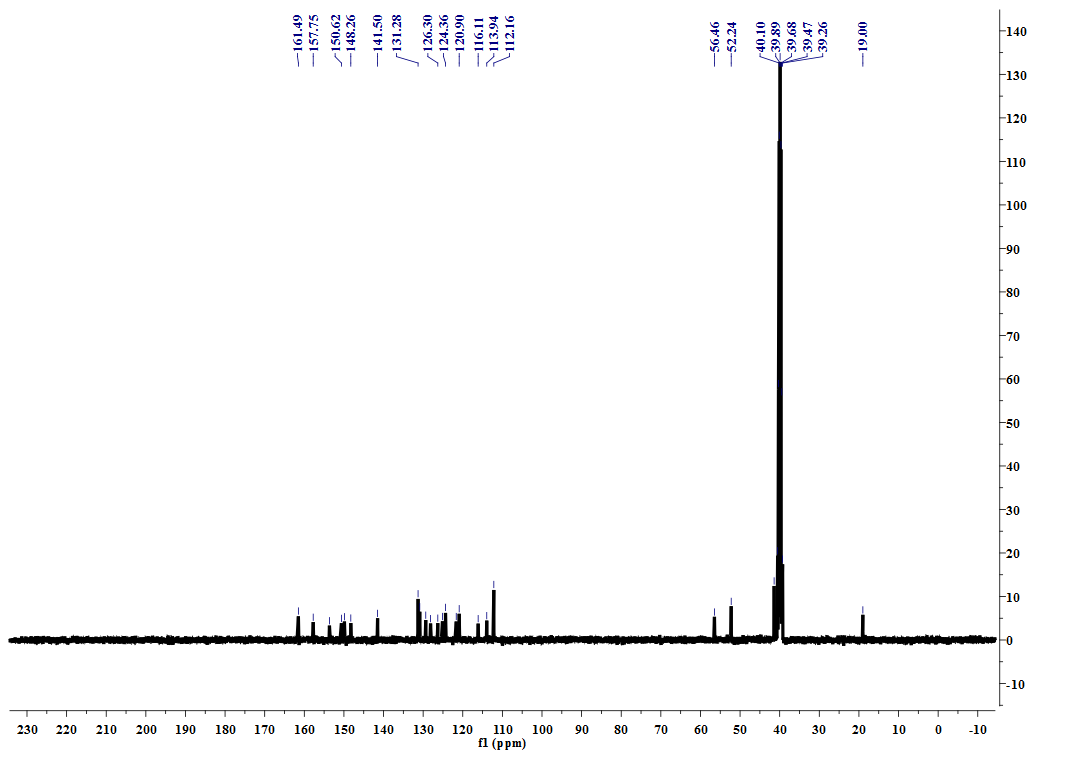


**Supplementary Figure 12.** The ^13^C NMR spectroscopy of **III** in DMSO-d_6_

### HRMS spectroscopies of 4-6


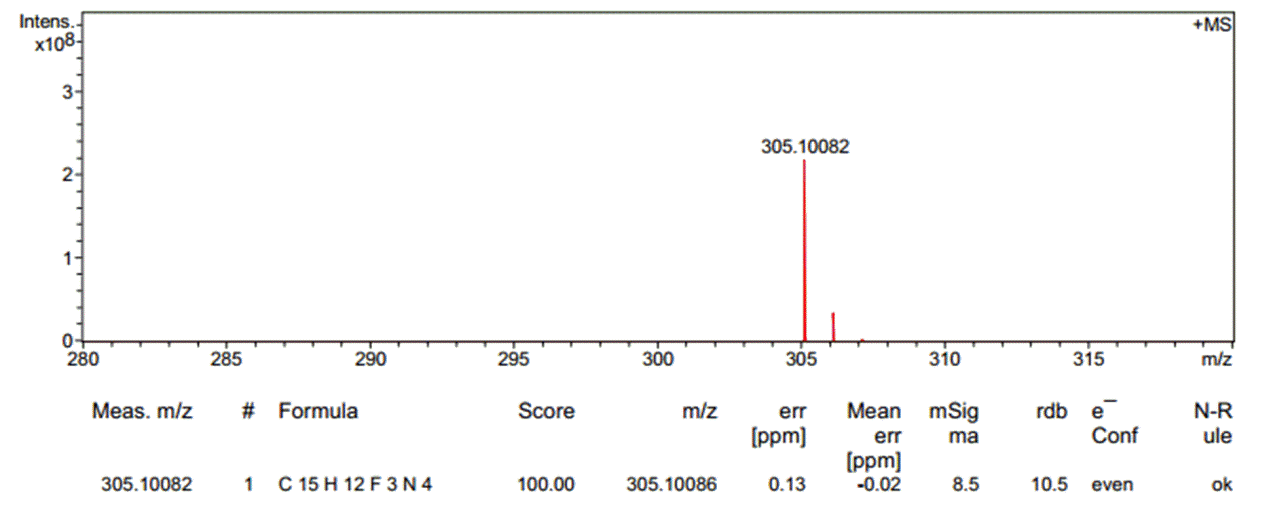

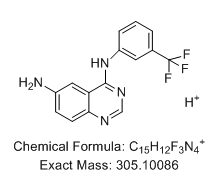


**Supplementary Figure 13.** The HR-MS spectroscopy of **4**


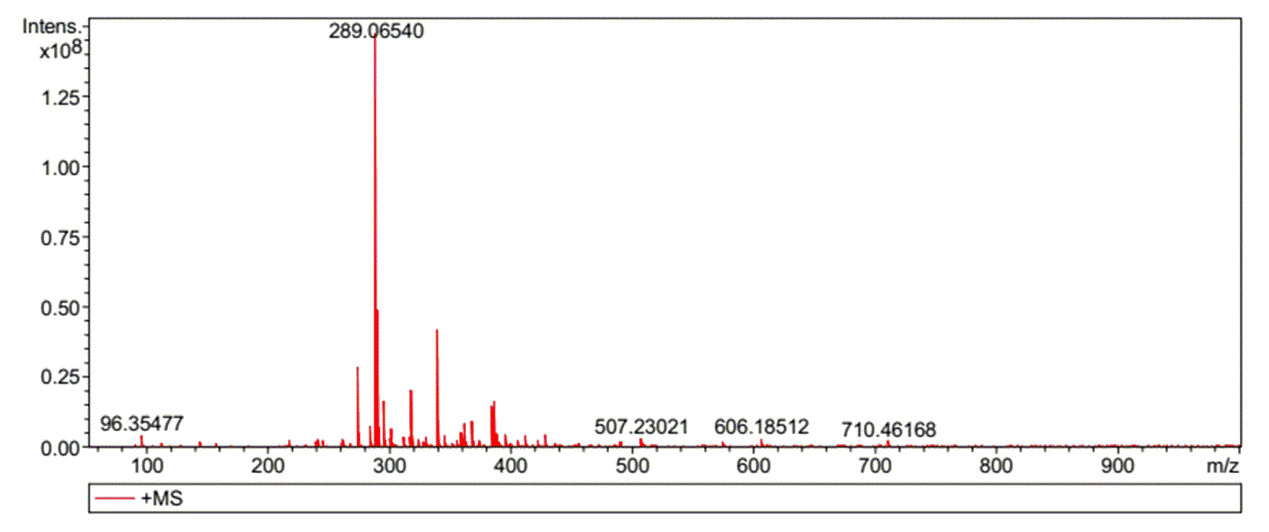

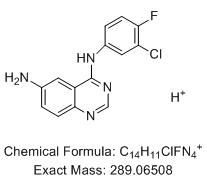


**Supplementary Figure 14.** The HR-MS spectroscopy of **5**


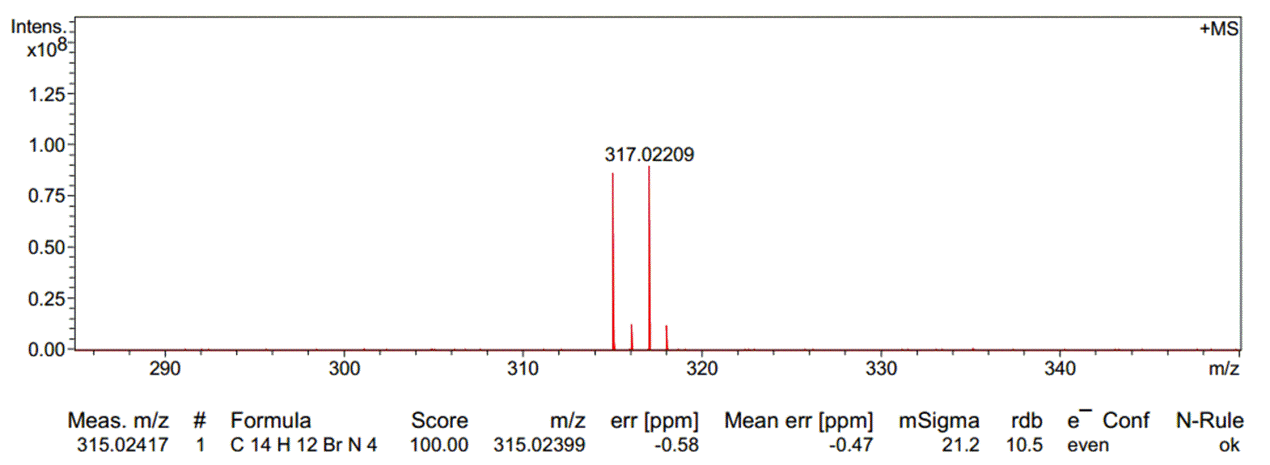

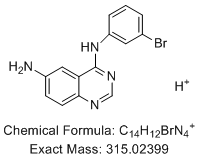


**Supplementary Figure 15.** The HR-MS spectroscopy of **6**

### HRMS spectroscopies of I-III


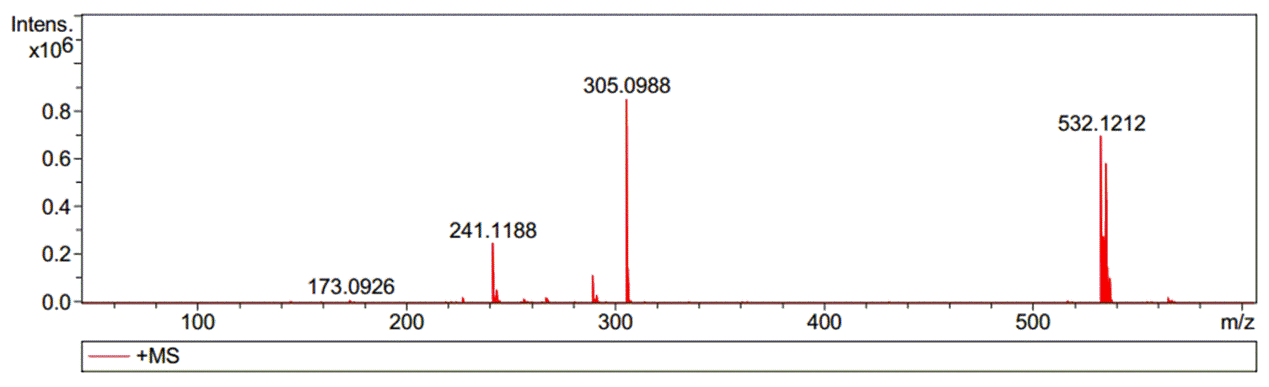

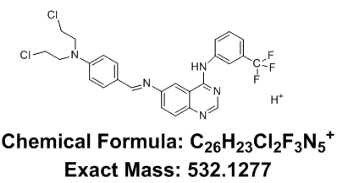


**Supplementary Figure 16.** The HR-MS spectroscopy of **I**


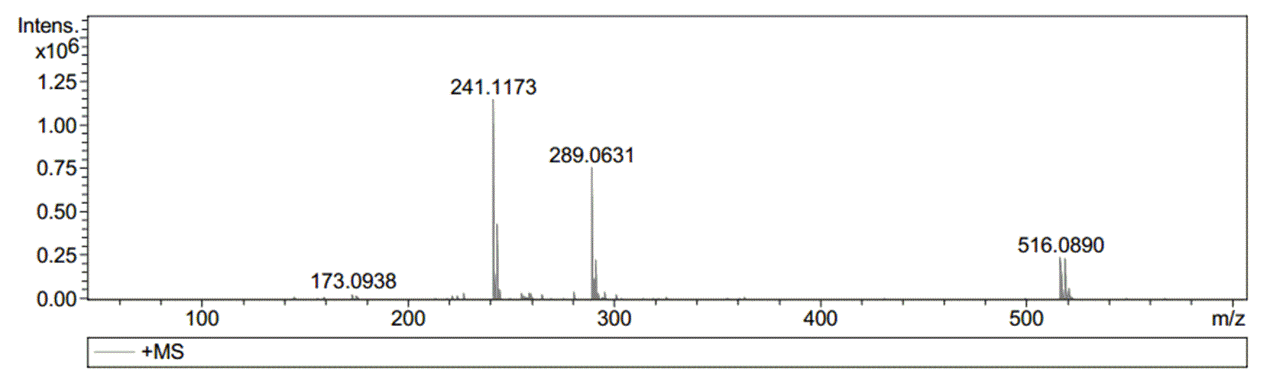

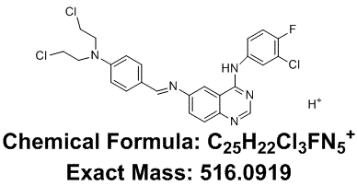


**Supplementary Figure 17.** The HR-MS spectroscopy of **II**

**Fig.**


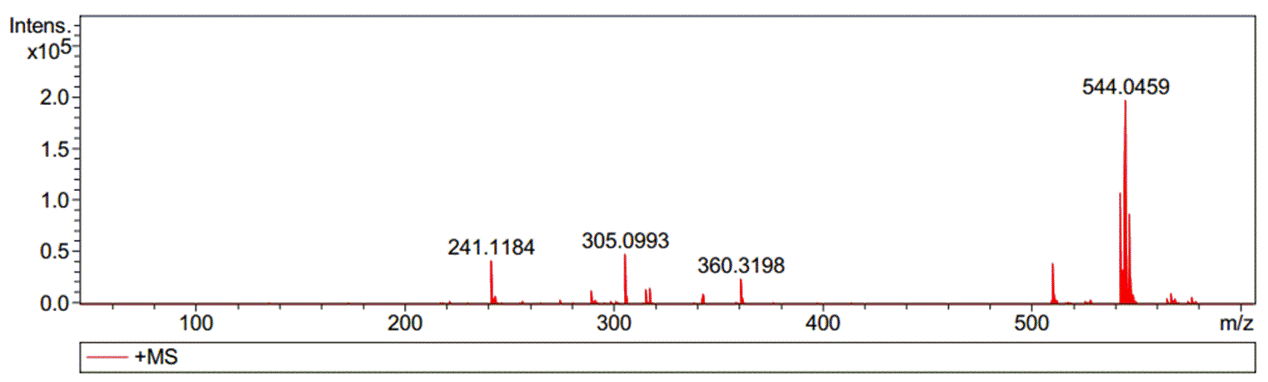

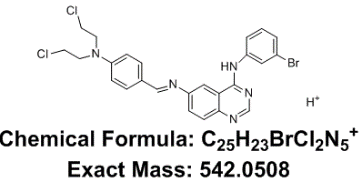

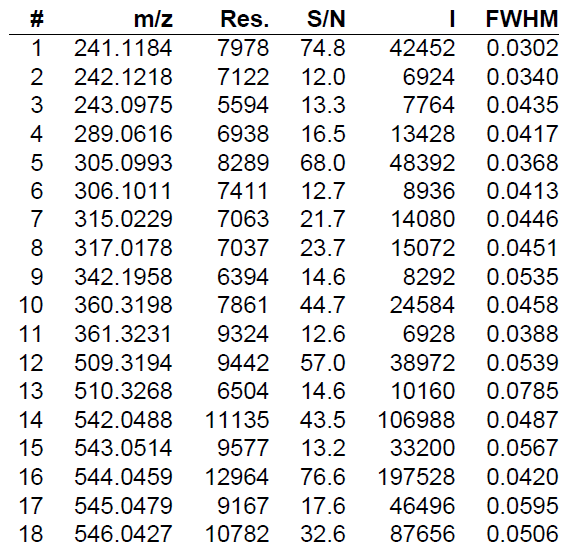


**Supplementary Figure 18.** The HR-MS spectroscopy of **III**

### FT-IR spectroscopies of 4-5


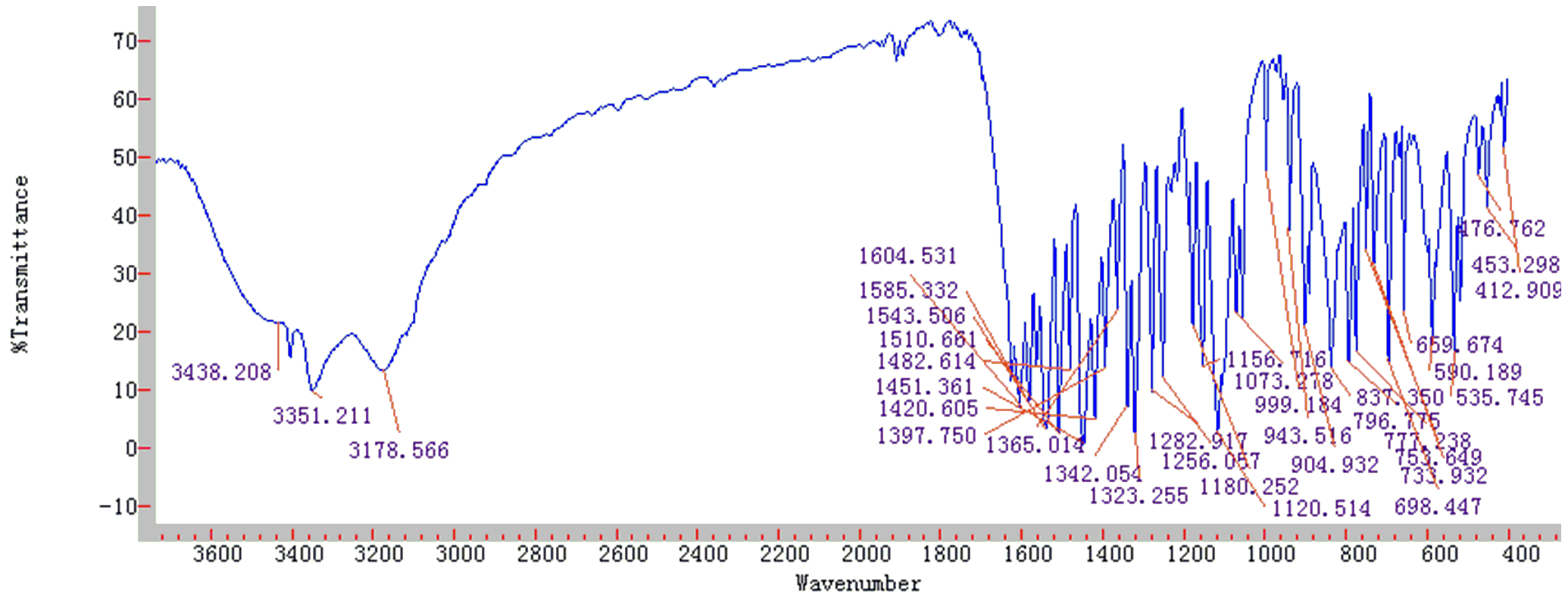

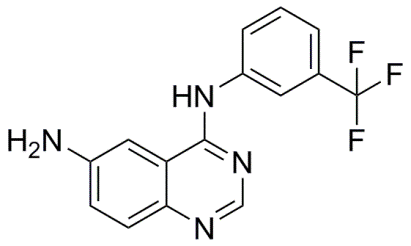


**Supplementary Figure 19.** The FT-IR spectroscopy of **4**


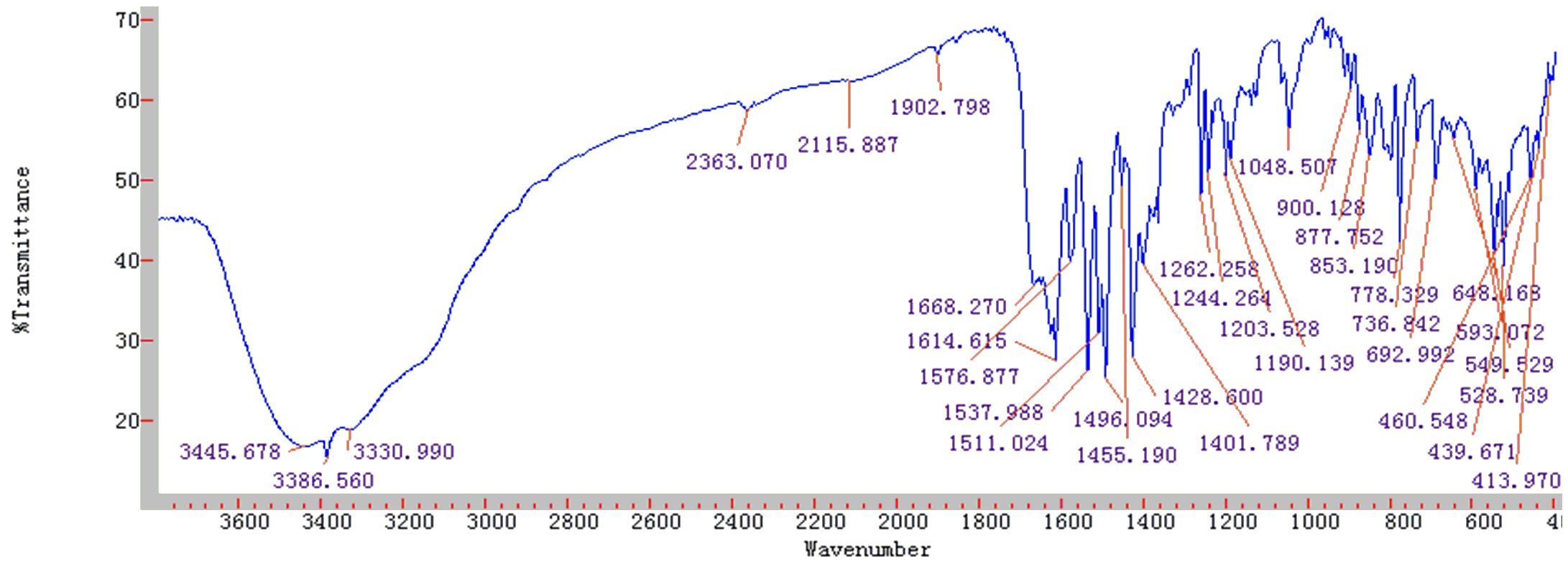

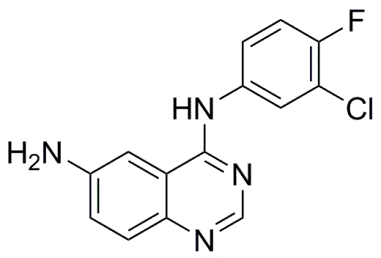


**Supplementary Figure 20.** The FT-IR spectroscopy of **5**


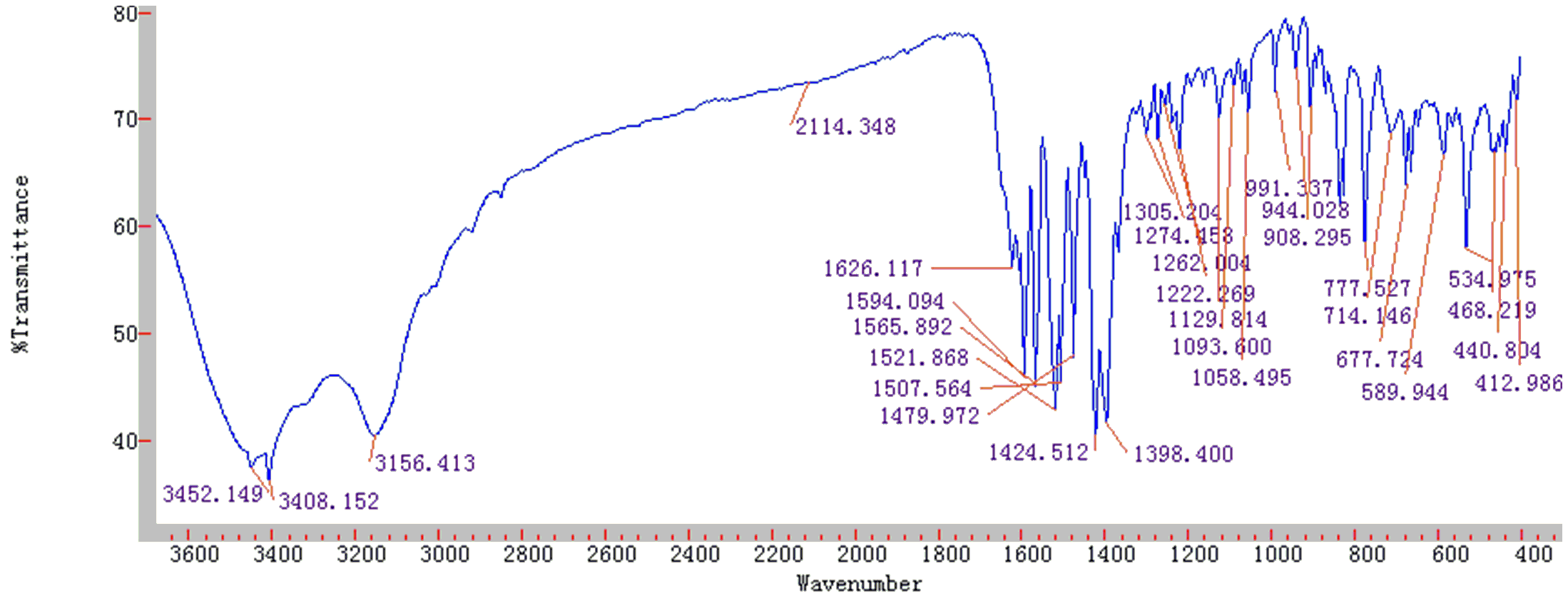

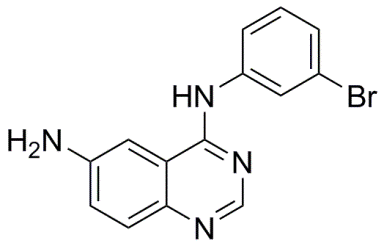


**Supplementary Figure 21.** The FT-IR spectroscopy of **6**

### FT-IR spectroscopies of I-III


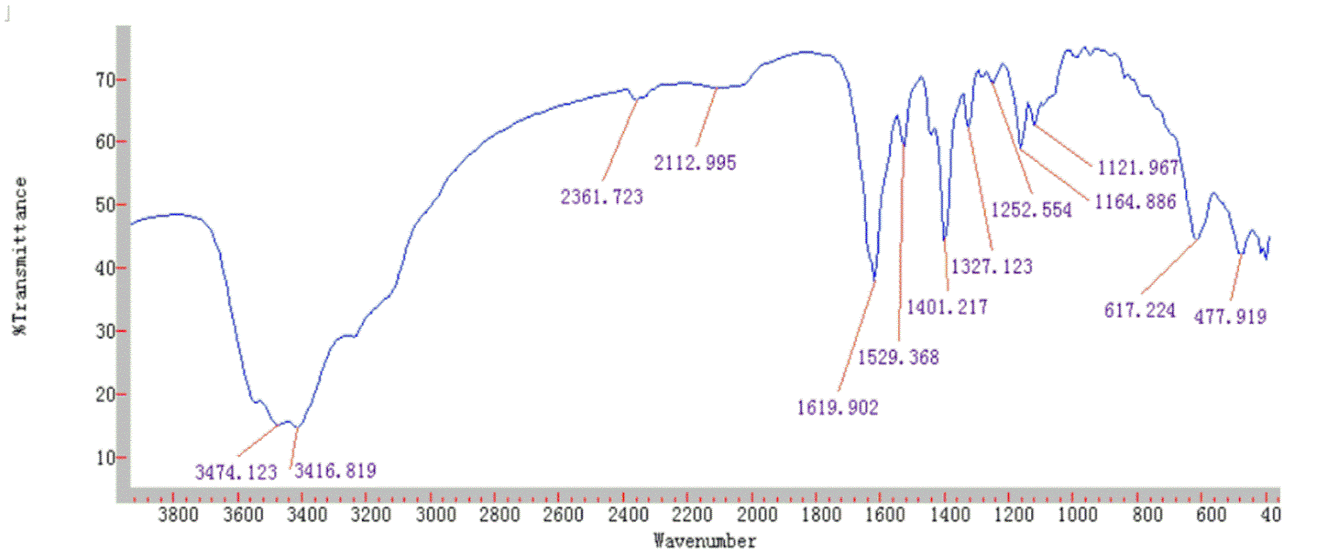

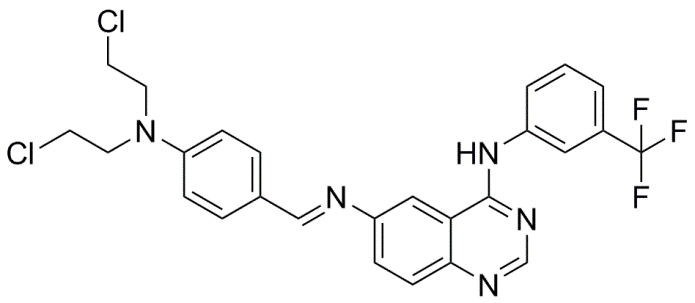


**Supplementary Figure 22.** The FT-IR spectroscopy of **I**


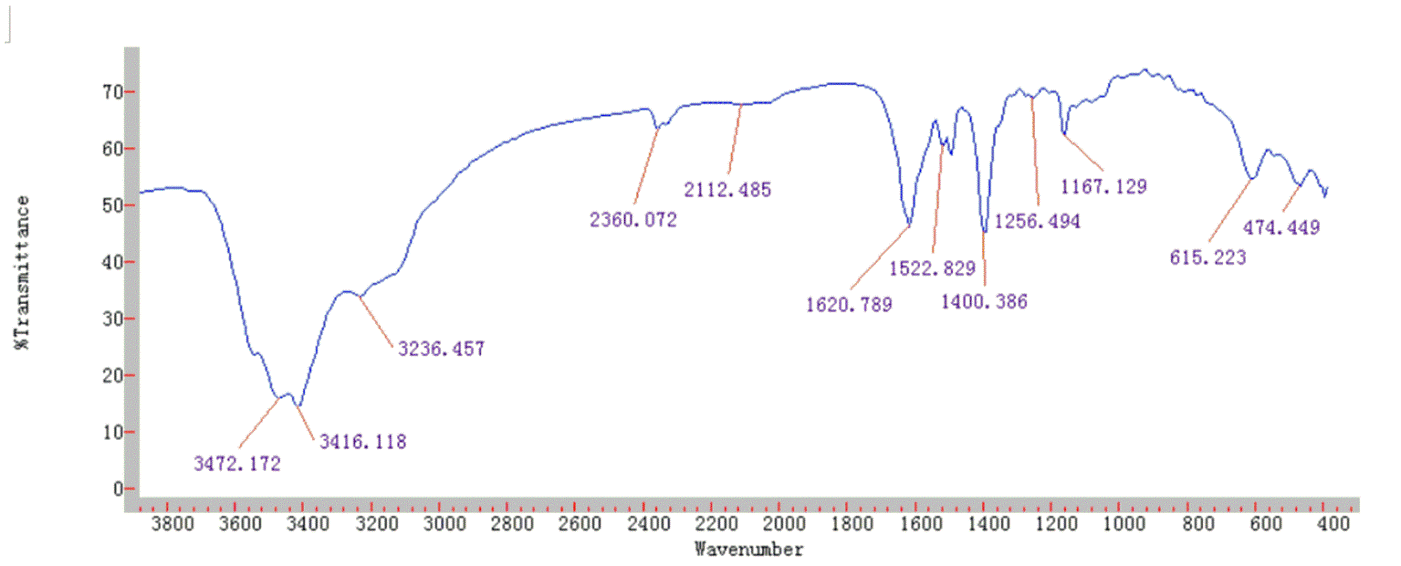

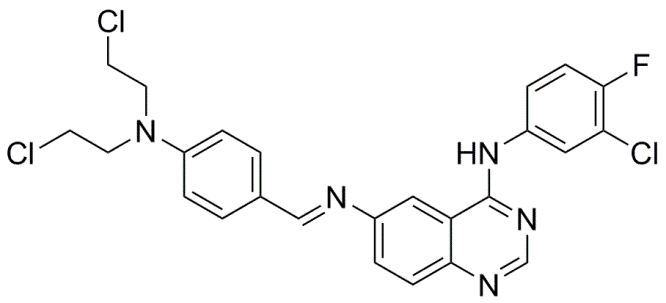


**Supplementary Figure 23.** The FT-IR spectroscopy of **II**


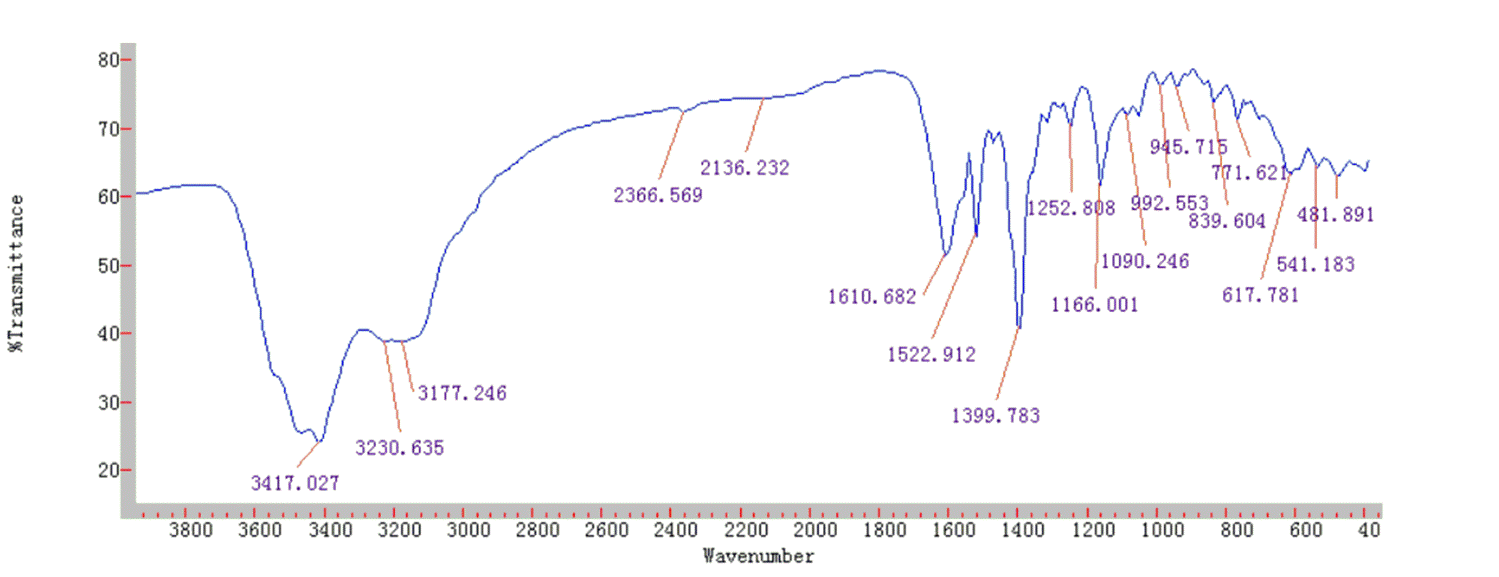

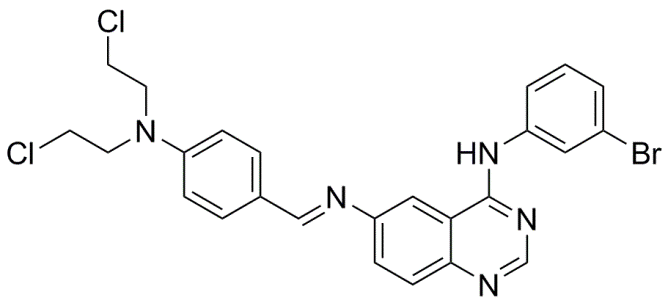


**Supplementary Figure 24.** The FT-IR spectroscopy of **III**

### X-Ray data for compound 5 and 6


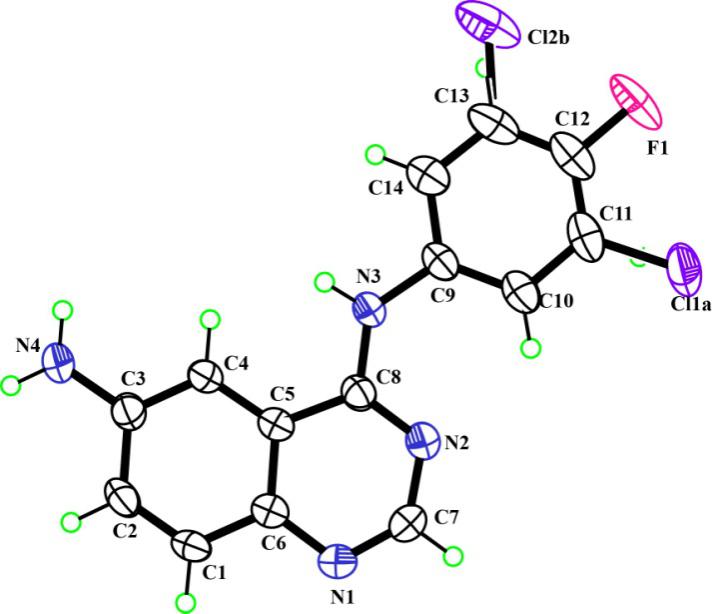


**Supplementary Figure 25.** The molecular structure of **5** (ellipsoid chance 30%)


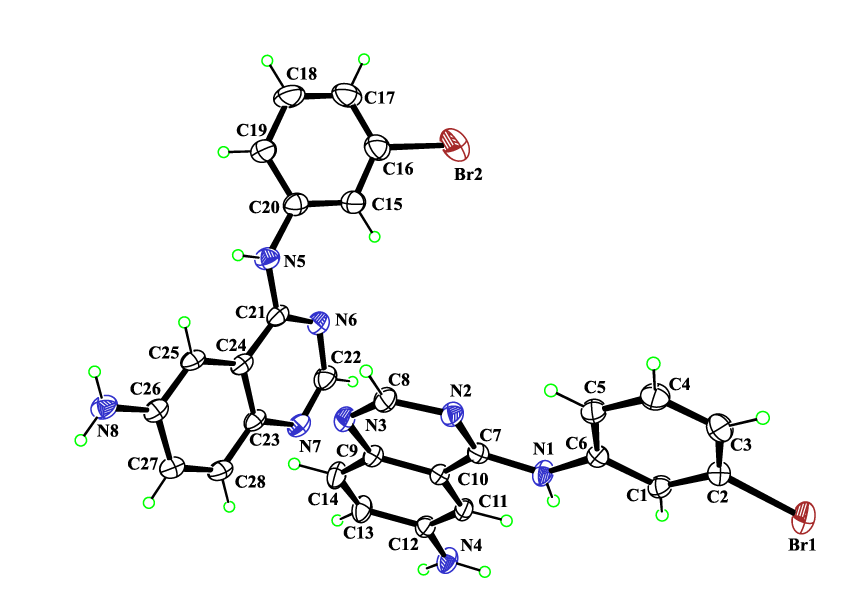


**Supplementary Figure 26.** The molecular structure of **6** (ellipsoid chance 30%)

### X-Ray data for compound II

**
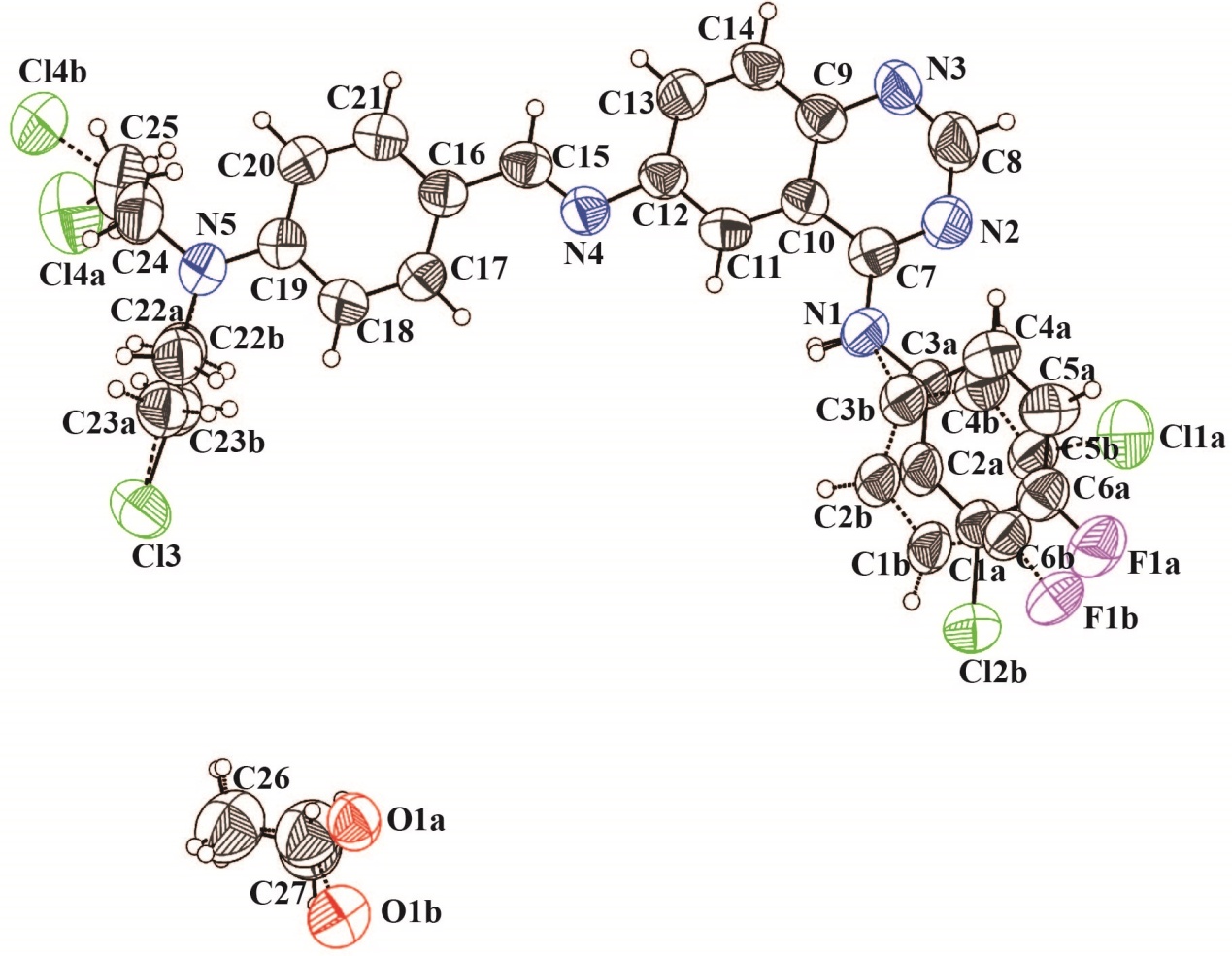
**

**Supplementary Figure 27.** The molecular structure of **II** (ellipsoid chance 30%)


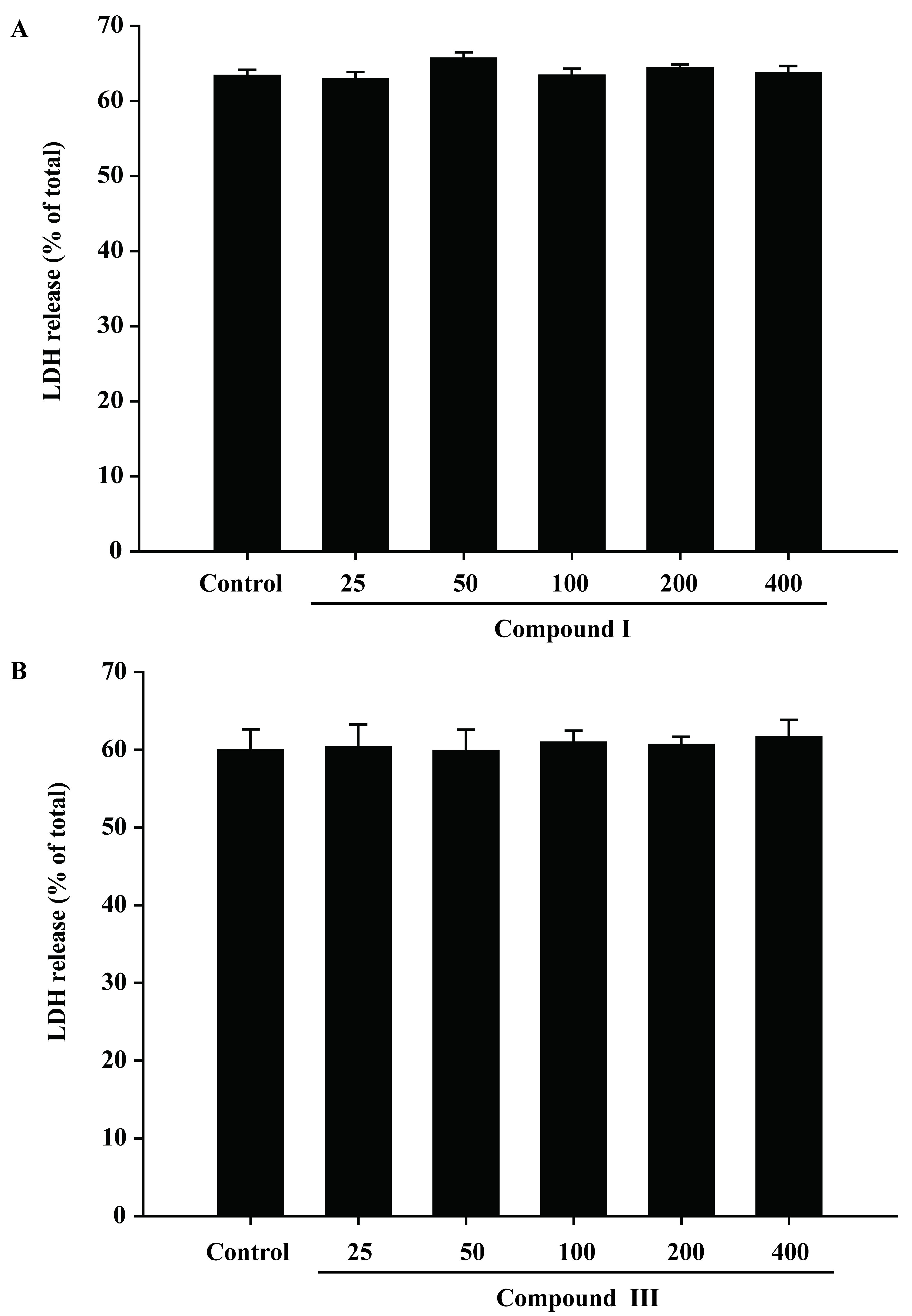


**Supplementary Figure 28.** The effects of compound I and III on LDH level in HepG2 cells. HepG2 cells treated with various concentrations of compound I and III for 24 h. (A) LDH release of HepG2 after exposure of compound I. (B) LDH release of HepG2 after exposure of compound III. Data were presented as mean ± SD. of three independent experiments.


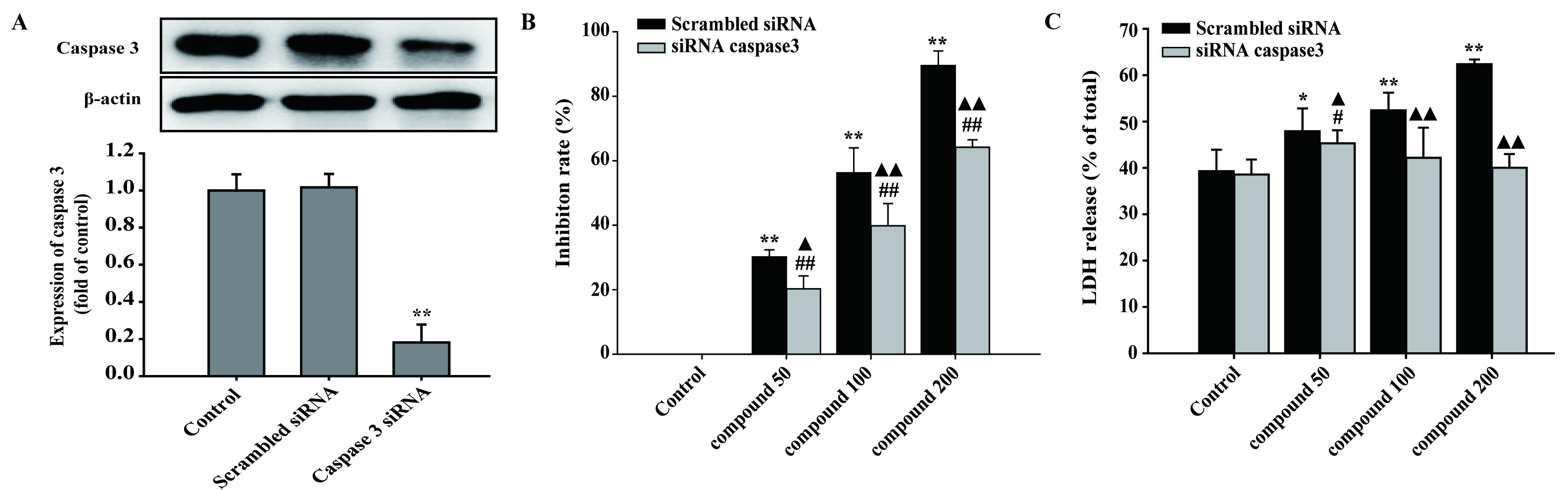


**Supplementary Figure 29.** Caspase 3 knockdown markedly abolished the inhibitory effects of compound II in HepG2 cells. (A) Representative western blot and quantification were shown for caspase 3 siRNA. (B) Inhibiton rate was determined in HepG2 cells transfected with or without caspase 3 siRNA. (J) LDH release was determined in HepG2 cells transfected with or without caspase 3 siRNA. The data were presented as the mean ± SD. *P < 0.05, **P < 0.01 versus control group; #P < 0.05, ##P < 0.01 versus scrambled siRNA control group; ▲P < 0.05, ▲▲P < 0.01 versus non-infected group.

## **Supplementary Tables**


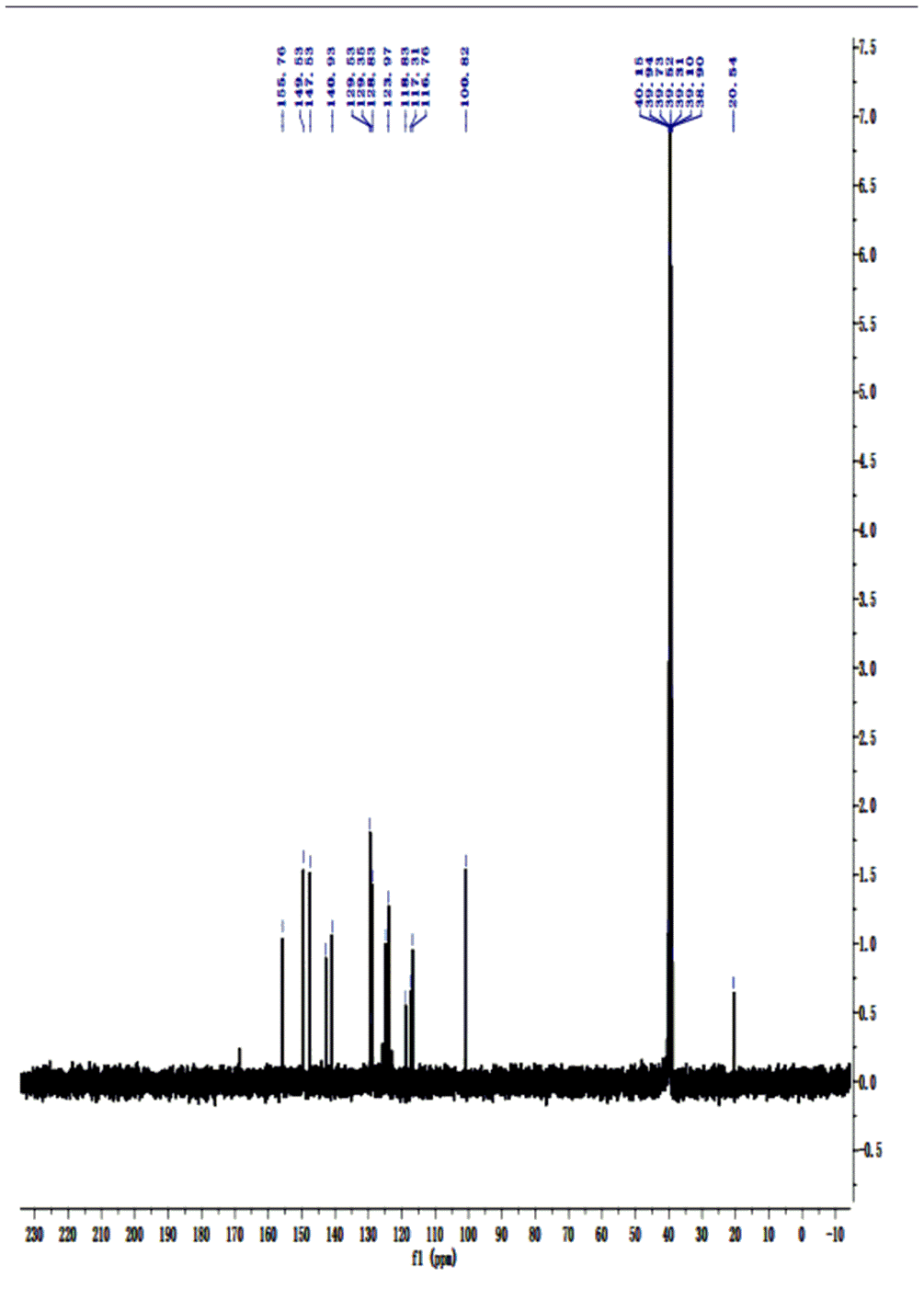

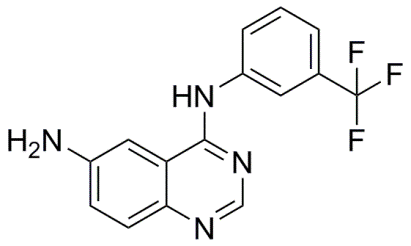


Supplementary Table **1.** Crystal data and structure refinement for **5** and **6**

| **Formula** | C_14_H_10_ClFN_4_(**5**) | C_14_H_11_BrN_4_(**6**) |
| --- | --- | --- |
| **Molecular weight** | 288.71 | 315.18 |
| **space group** | P-1 | P-1 |
| ***a* (A, )** | 7.3150(11) | 7.6072(14) |
| ***b* (A, )** | 9.1215(13) | 11.745(2) |
| ***c* (A, )** | 10.2561(15) | 15.841(3) |
| ***β* (°)** | 92.521(3) | 96.539(5) |
| ***V* (Å^3^)** | 633.85(16) | 1303.2(4) |
| ***Z*** | 2 | 4 |
| ***Dcalc (g cm-3)*** | 1.513 | 1.606 |
| ***Absorption corr***  ***Ψ-scan max., min*** | 0.9467,0.9354 | 0.6014,0.5446 |
| ***F(000)*** | 296 | 632 |
| ***Crystal size (mm)*** | 0.22×0.20×0.18 | 0.22×0.20×0.18 |
| ***θ range data collection*** | 1.99-25.31 | 1.39-25.20 |
| ***h,k,lmax*** | 8,10,12 | 9,14,18 |
| ***Goodness-of-fit on F2*** | 1.123 | 1.069 |
| ***R [I>2δ(I)]*** | 0.0839 | 0.0716 |
| ***wR2 (all data)*** | 0.1848 | 0.1306 |

Supplementary Table **2.** Selected bond lengths(Å) and angles (°) of **5** and **6**

| **5** | | **6** | |
| --- | --- | --- | --- |
| *Bond lengths* |  |  |  |
| C1-C2 | 1.369(6) | Br1-C2 | 1.903(4) |
| C1-C6 | 1.406(5) | Br2-C16 | 1.891(5) |
| C2-C3 | 1.405(6) | C1-C2 | 1.372(5) |
| C3-C4 | 1.377(5) | C1-C6 | 1.396(5) |
| C3-N4 | 1.399(5) | C2-C3 | 1.379(6) |
| C4-C5 | 1.395(5) | C3-C4 | 1.379(6) |
| C5-C6 | 1.412(5) | C4-C5 | 1.377(6) |
| C5-C8 | 1.443(5) | C5-C6 | 1.395(5) |
| C6-N1 | 1.380(5) | C6-N1 | 1.408(5) |
| C7-N1 | 1.308(5) | C7-N2 | 1.328(5) |
| C7-N2 | 1.365(5) | C7-N1 | 1.359(5) |
| C8-N2 | 1.320(5) | C7-C10 | 1.435(5) |
| C8-N3 | 1.372(5) | C8-N3 | 1.303(5) |
| C9-N3 | 1.404(5) | C8-N2 | 1.354(5) |
| C9-C10 | 1.406(6) | C9-N3 | 1.379(5) |
| C9-C14 | 1.414(6) | C11-C12 | 1.375(5) |
| C10-C11 | 1.392(6) | C12-N4 | 1.389(5) |
| C11-C12 | 1.340(7) | C12-C13 | 1.413(5) |
| C11-Cl1A | 1.685(5) | C20-N5 | 1.410(6) |
| C12-F1 | 1.365(5) | C21-N6 | 1.317(5) |
| C12-C13 | 1.386(7) | C21-N5 | 1.373(5) |
| C13-C14 | 1.365(6) | C21-C24 | 1.429(6) |
| C13-Cl1B | 1.629(7) | C22-N7 | 1.292(6) |
|  |  | C22-N6 | 1.354(5) |
|  |  | C23-N7 | 1.375(5) |
|  |  | C23-C28 | 1.402(6) |
|  |  | C25-C26 | 1.377(6) |
|  |  | C26-N8 | 1.386(5) |
|  |  | C26-C27 | 1.419(5) |

| *Bond angles* |  |  |  |
| --- | --- | --- | --- |
| C2-C1-C6 | 121.0(4) | C2-C1-C6 | 119.4(3) |
| C1-C2-C3 | 121.1(4) | C1-C2-C3 | 122.3(4) |
| C4-C3-N4 | 120.8(4) | C1-C2-Br1 | 118.6(3) |
| C4-C3-C2 | 118.6(4) | C3-C2-Br1 | 119.1(3) |
| N4-C3-C2 | 120.6(4) | C4-C3-C2 | 117.5(4) |
| C3-C4-C5 | 121.3(4) | C3-C4-C5 | 122.4(4) |
| C4-C5-C6 | 120.1(3) | C4-C5-C6 | 118.9(4) |
| C4-C5-C8 | 125.1(3) | C1-C6-C5 | 119.5(3) |
| C6-C5-C8 | 114.8(3) | C1-C6-N1 | 115.7(3) |
| N1-C6-C1 | 119.2(3) | C5-C6-N1 | 124.7(3) |
| N1-C6-C5 | 122.9(4) | N2-C7-N1 | 119.7(3) |
| C1-C6-C5 | 117.9(4) | N2-C7-C10 | 121.8(3) |
| N1-C7-N2 | 128.7(4) | N1-C7-C10 | 118.5(3) |
| N2-C8-N3 | 119.7(3) | N3-C8-N2 | 129.5(4) |
| N2-C8-C5 | 122.4(3) | N3-C9-C14 | 119.8(3) |
| N3-C8-C5 | 117.9(3) | N3-C9-C10 | 121.6(3) |
| N3-C9-C10 | 125.1(4) | C14-C9-C10 | 118.6(3) |
| N3-C9-C14 | 115.9(4) | C11-C10-C9 | 119.2(3) |
| C10-C9-C14 | 119.0(4) | C11-C10-C7 | 124.9(3) |
| C11-C10-C9 | 118.8(4) | C9-C10-C7 | 115.9(3) |
| C12-C11-C10 | 120.8(4) | C12-C11-C10 | 121.2(3) |
| C12-C11-Cl1A | 118.1(3) | C11-C12-N4 | 121.6(3) |
| C10-C11-Cl1A | 121.0(4) | C11-C12-C13 | 118.6(3) |
| C11-C12-F1 | 120.7(4) | N4-C12-C13 | 119.7(3) |
| F1-C12-C13 | 117.5(4) | C13-C14-C9 | 121.4(4) |
| C14-C13-C12 | 119.4(4) | C20-C15-C16 | 118.6(4) |
| C14-C13-Cl1B | 125.1(5) | C17-C16-C15 | 122.1(5) |
| C12-C13-Cl1B | 115.4(4) | C17-C16-Br2 | 119.2(4) |
| C13-C14-C9 | 120.2(5) | C15-C16-Br2 | 118.7(4) |
| C7-N1-C6 | 114.9(3) | C16-C17-C18 | 119.1(5) |
| C8-N2-C7 | 116.2(3) | C17-C18-C19 | 120.0(5) |
| C8-N3-C9 | 131.0(3) | C18-C19-C20 | 120.7(5) |
|  |  | N7-C23-C28 | 119.9(3) |
|  |  | N7-C23-C24 | 121.7(4) |
|  |  | C27-C28-C23 | 121.7(4) |
|  |  | C7-N1-C6 | 131.9(3) |
|  |  | C7-N2-C8 | 115.9(3) |
|  |  | C8-N3-C9 | 115.1(3) |
|  |  | C21-N5-C20 | 130.2(4) |
|  |  | C21-N6-C22 | 115.9(4) |
|  |  | C22-N7-C23 | 115.3(3) |

Supplementary Table **3.** Crystal data and structure refinement for **II**

| **Formula** | C_27_H_26_C_l3_FN_5_O |
| --- | --- |
| **Molecular weight** | 561.88 |
| **space group** | P2(1)/n |
| ***a* (A, )** | 9.482(13) |
| ***b* (A, )** | 16.266(18) |
| ***c* (A, )** | 17.36(2) |
| ***β* (°)** | 101.10(4) |
| ***V* (Å^3^)** | 2628(6) |
| ***Z*** | 4 |
| ***D*_calc_ (g cm^-3^)** | 1.420 |
| **Absorption corr Ψ-scan max., min** | 0.9336,0.9197 |
| ***F*(000)** | 1164 |
| **Crystal size (mm)** | 0.22×0.20×0.18 |
| ***θ* range data collection** | 1.73-25.27 |
| **h,k,lmax** | 11,16,20 |
| **Goodness-of-fit on *F*^2^** | 0.953 |
| ***R* [*I>*2δ(*I*)]** | 0.1661 |
| ***wR*^2^ (all data)** | 0.2154 |

Supplementary Table **4.** Selected bond lengths(Å) and angles (°) of **II**

| *Bond lengths* |  |  |  |  |  |
| --- | --- | --- | --- | --- | --- |
| C1A-C2A | 1.3900 | C1A-C6A | 1.3900 | C1A-Cl1A | 1.706(3) |
| C2A-C3A | 1.3900 | C3A-C4A | 1.3900 | C3A-N1 | 1.403(5) |
| C4A-C5A | 1.3900 | C5A-C6A | 1.3900 | C6A-F1A | 1.310(4) |
| C1B-C2B | 1.3900 | C1B-C6B | 1.3900 | C1B-Cl1B | 1.669(7) |
| C2B-C3B | 1.3900 | C3B-N1 | 1.359(8) | C3B-C4B | 1.3900 |
| C4B-C5B | 1.3900 | C5B-C6B | 1.3900 | C6B-F1B | 1.308(8) |
| C7-N2 | 1.307(6) | C7-N1 | 1.353(6) | C7-C10 | 1.425(7) |
| C8-N3 | 1.287(7) | C8-N2 | 1.346(7) | C9-C14 | 1.366(7) |
| C9-N3 | 1.370(6) | C9-C10 | 1.404(6) | C10-C11 | 1.387(7) |
| C11-C12 | 1.360(6) | C12-C13 | 1.394(7) | C12-N4 | 1.403(6) |
| C13-C14 | 1.344(7) | C15-N4 | 1.239(6) | C15-C16 | 1.446(7) |
| C16-C21 | 1.385(7) | C16-C17 | 1.383(7) | C17-C18 | 1.350(7) |
| C18-C19 | 1.378(7) | C19-N5 | 1.371(7) | C19-C20 | 1.392(7) |
| C20-C21 | 1.351(7) | C22-N5 | 1.395(7) | C22-C23 | 1.477(8) |
| C23-Cl3B | 1.535(9) | C23-Cl3A | 1.660(6) | C24A-C25A | 1.499(9) |
| C24A-N5 | 1.536(8) | C25A-Cl2 | 1.815(8) | C24B-C25B | 1.501(12) |
| C24B-N5 | 1.550(11) | C25B-Cl2 | 1.808(11) | C27A-C26 | 1.360(10) |
| C27A-O1A | 1.422(10) | C27B-C26 | 1.370(13) | C27B-O1B | 1.427(14) |

| *Bond angles* |  |  |  |  |  |
| --- | --- | --- | --- | --- | --- |
| C2A-C1A-C6A | 120.0 | C2A-C1A-Cl1A | 120.6(2) | C6A-C1A-Cl1A | 119.43(19) |
| C1A-C2A-C3A | 120.0 | C4A-C3A-C2A | 120.0 | C4A-C3A-N1 | 121.9(3) |
| C2A-C3A-N1 | 117.9(3) | C3A-C4A-C5A | 120.0 | C6A-C5A C4A | 120.0 |
| F1A-C6A-C5A | 120.5(3) | F1A-C6A-C1A | 119.5(3) | C5A-C6A-C1A | 120.0 |
| C2B-C1B-C6B | 120.0 | C2B-C1B-Cl1B | 117.4(4) | C6B-C1B-Cl1B | 122.6(4) |
| C3B-C2B-C1B | 120.0 | N1-C3B-C2B | 116.4(5) | N1-C3B-C4B | 121.6(5) |
| C2B-C3B-C4B | 120.0 | C5B-C4B-C3B | 120.0 | C6B-C5B-C4B | 120.0 |
| F1B-C6B-C5B | 119.8(5) | F1B-C6B-C1B | 120.2(5) | C5B-C6B-C1B | 120.0 |
| N2-C7-N1 | 117.6(5) | N2-C7-C10 | 121.6(5) | N1-C7-C10 | 120.8(5) |
| N3-C8-N2 | 130.2(6) | C14-C9-N3 | 118.9(5) | C14-C9-C10 | 118.8(5) |
| N3-C9-C10 | 122.3(5) | C11-C10-C9 | 119.7(5) | C11-C10-C7 | 124.5(5) |
| C9-C10-C7 | 115.7(5) | C12-C11-C10 | 120.6(5) | C11-C12-C13 | 118.4(5) |
| C11-C12-N4 | 116.7(5) | C13-C12-N4 | 124.8(5) | C14-C13-C12 | 121.8(5) |
| C13-C14-C9 | 120.6(5) | N4-C15-C16 | 123.7(5) | C21-C16-C17 | 116.2(5) |
| C21-C16-C15 | 123.8(5) | C17-C16-C15 | 120.0(5) | C18-C17-C16 | 122.3(5) |
| C17-C18-C19 | 120.7(5) | N5-C19-C18 | 121.9(5) | N5-C19-C20 | 120.0(5) |
| C18-C19-C20 | 118.2(5) | C21-C20-C19 | 120.0(5) | C20-C21-C16 | 122.6(5) |
| N5-C22-C23 | 116.2(5) | C22-C23-Cl3B | 135.8(7) | C22-C23-Cl3A | 116.6(5) |
| Cl3B-C23-Cl3A | 70.1(6) | C25A-C24A-N5 | 104.4(5) | C24A-C25A-Cl2 | 105.1(5) |
| C25B-C24B-N5 | 102.5(8) | C24B-C25B-Cl2 | 106.1(8) | C25B-Cl2-C25A | 40.5(6) |
| C7-N1-C3B | 144.6(6) | C7-N1-C3A | 125.1(4) | C3B-N1-C3A | 19.6(6) |
| C7-N2-C8 | 116.1(5) | C8-N3-C9 | 114.1(5) | C15-N4-C12 | 119.6(5) |
| C19-N5-C22 | 122.1(5) | C19-N5-C24A | 121.8(5) | C22-N5-C24A | 114.3(5) |
| C19-N5-C24B | 119.3(10) | C22-N5-C24B | 109.7(10) | C24A-N5-C24B | 44.9(6) |
| C26-C27A-O1A | 114.2(8) | C26-C27B-O1B | 114.7(14) | C27A-C26-C27B | 21.6(19) |
