# Supplementary material for: Synthesis of a New Phenyl Chlormethine-Quinazoline Derivative, a Potential Anti-Cancer Agent, Induced Apoptosis in Hepatocellular Carcinoma Through Mediating Sirt1/Caspase 3 Signaling Pathway
Source: Front Pharmacol. 2020 Jun 26;11:911. doi: 10.3389/fphar.2020.00911 (PMC7332554; doi:10.3389/fphar.2020.00911)

Figure 4

Bax:

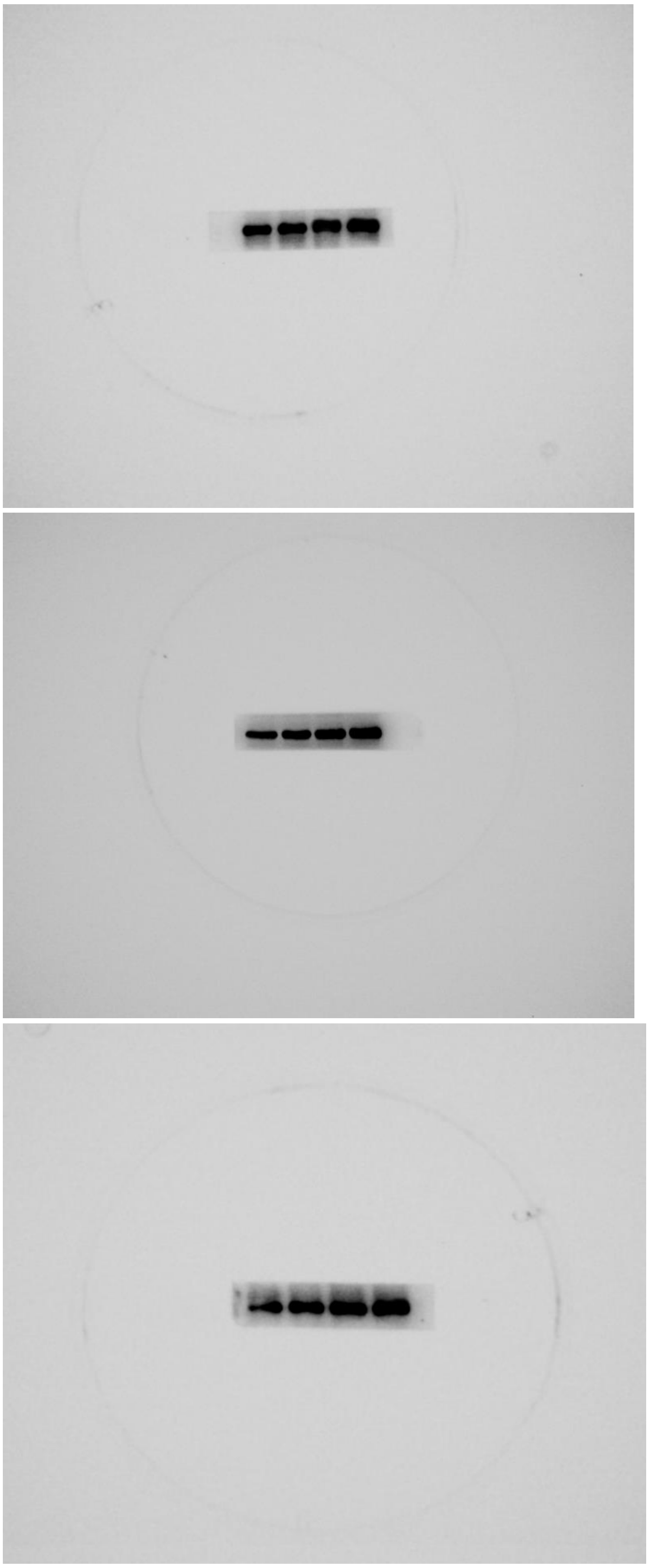

Bcl-2:

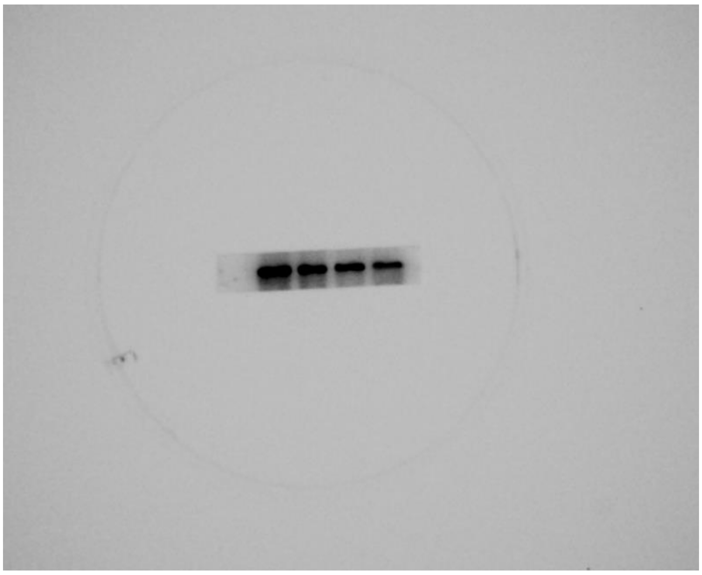

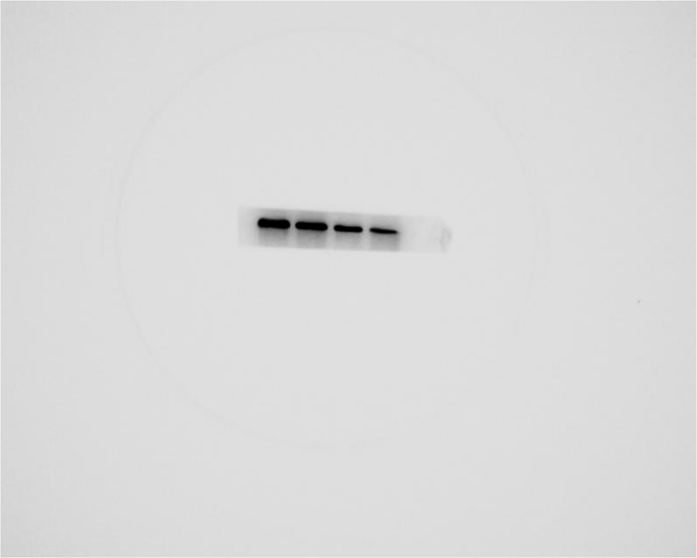

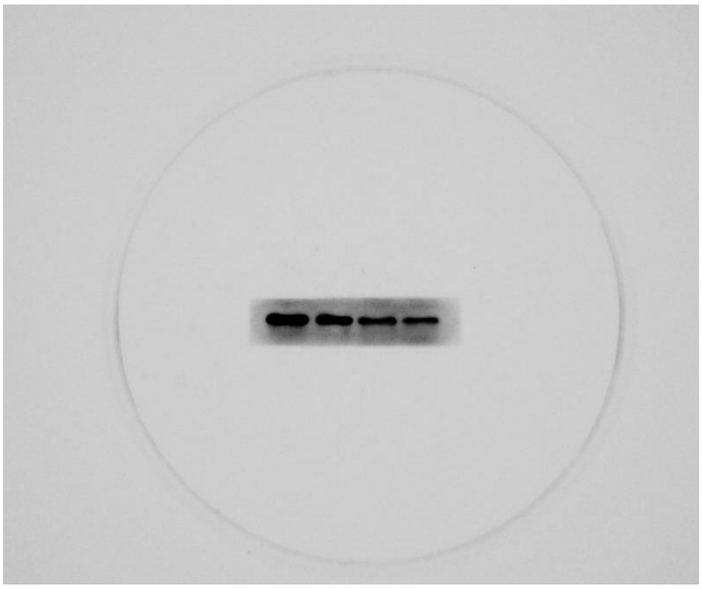

Active-caspase 3:

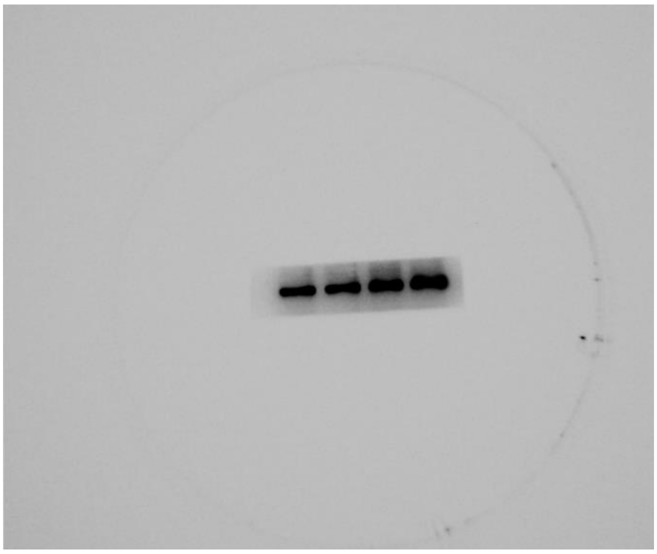

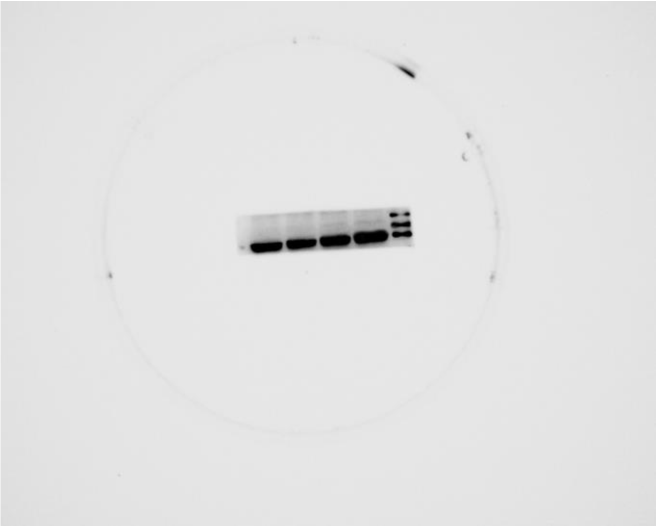

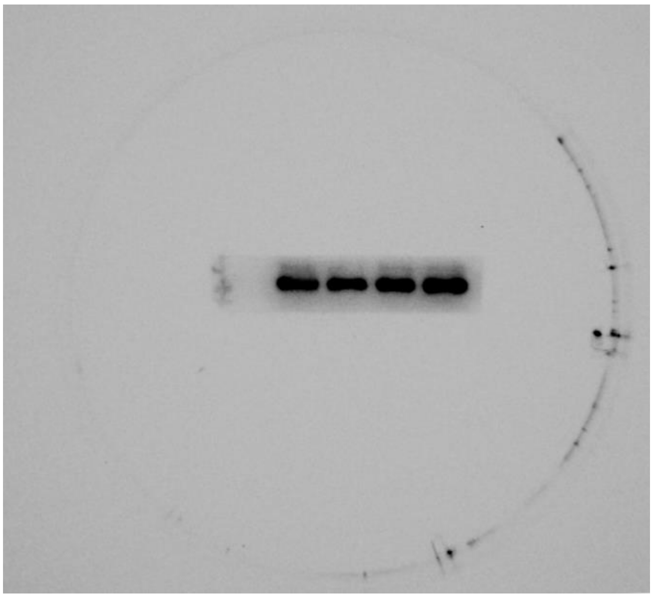

Sirt1:

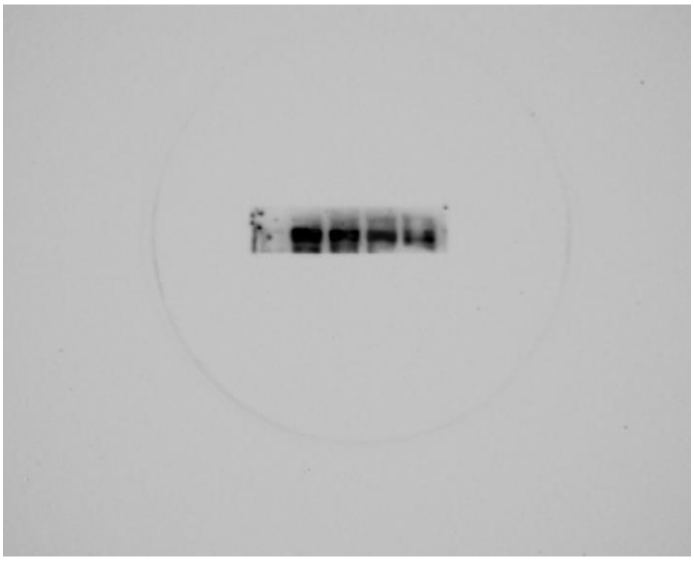

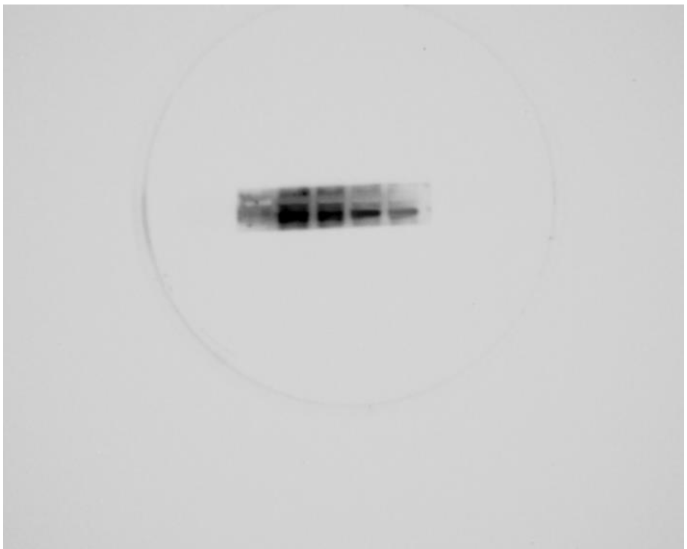

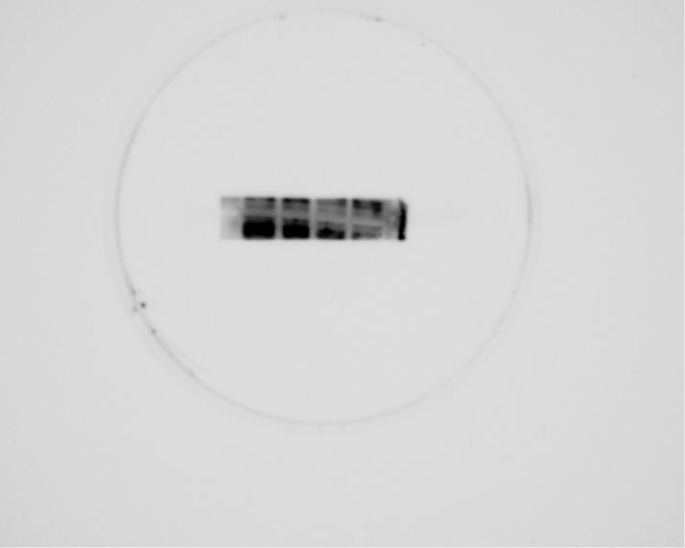

PGC-1α:

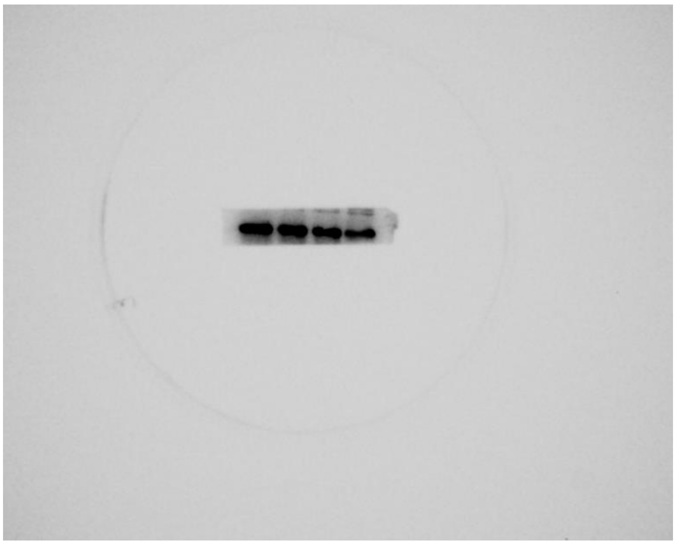

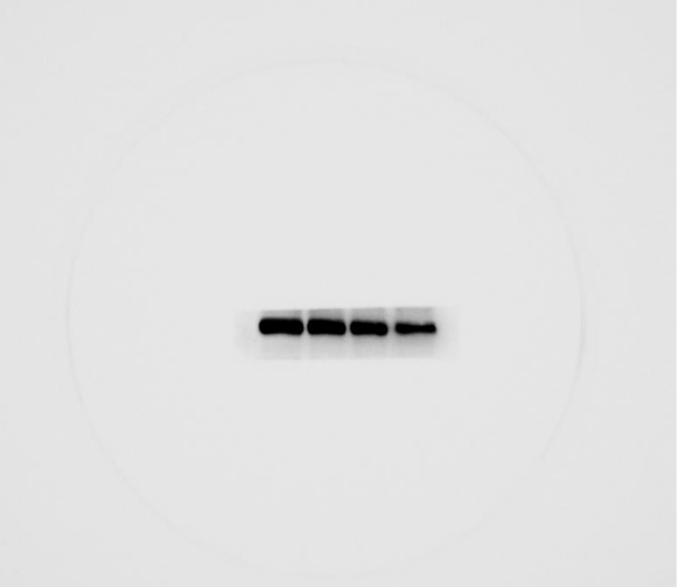

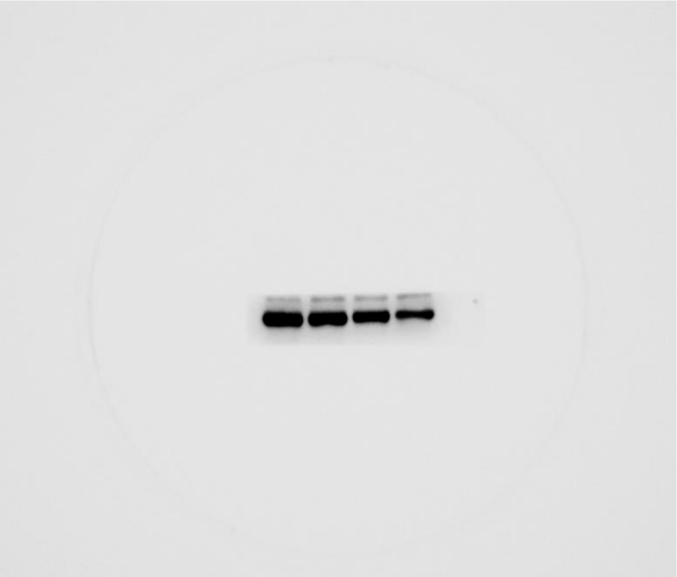

β-actin:

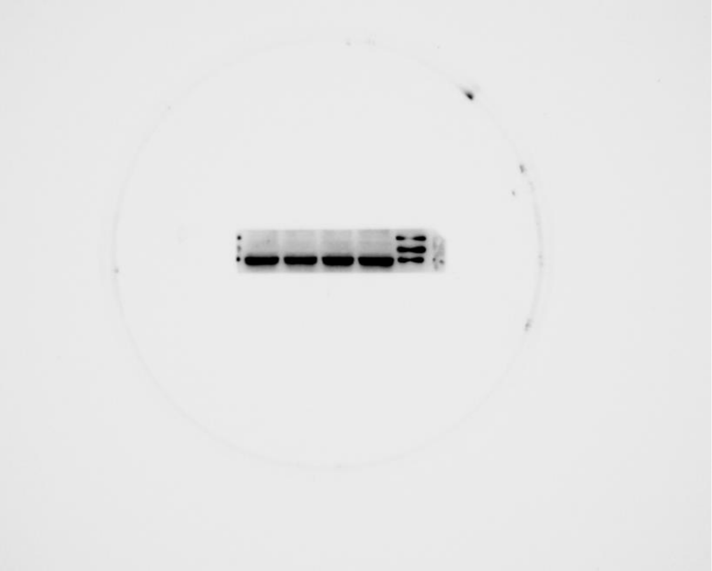

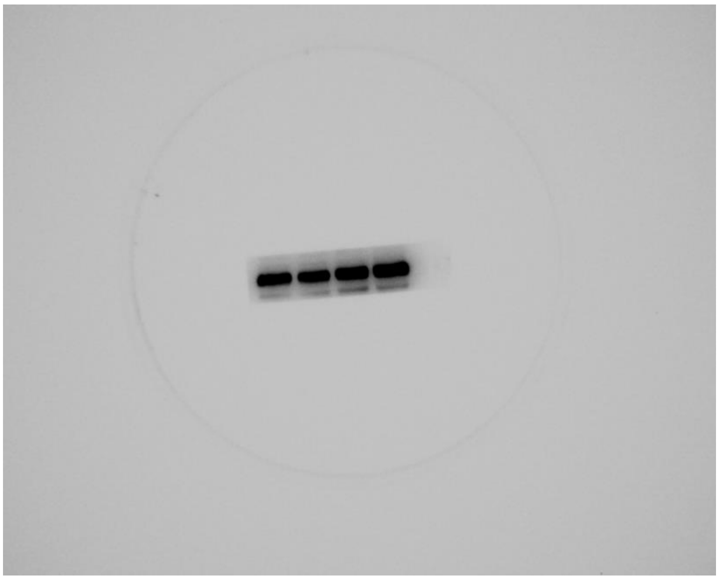

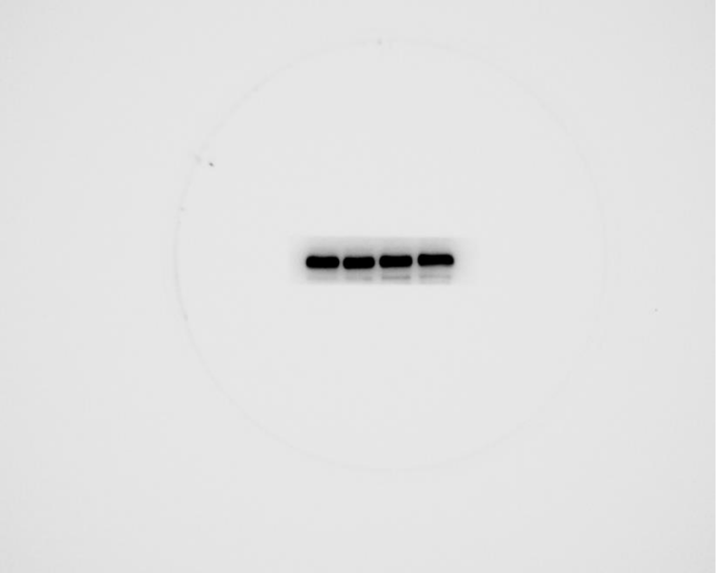

Figure 7

Bax:

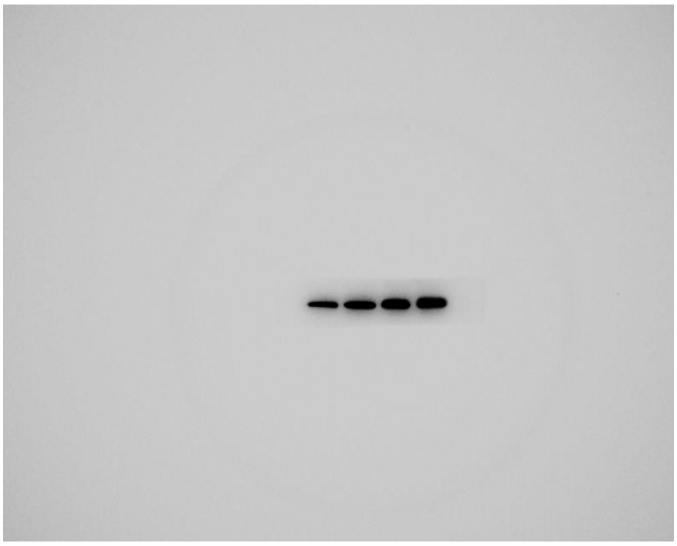

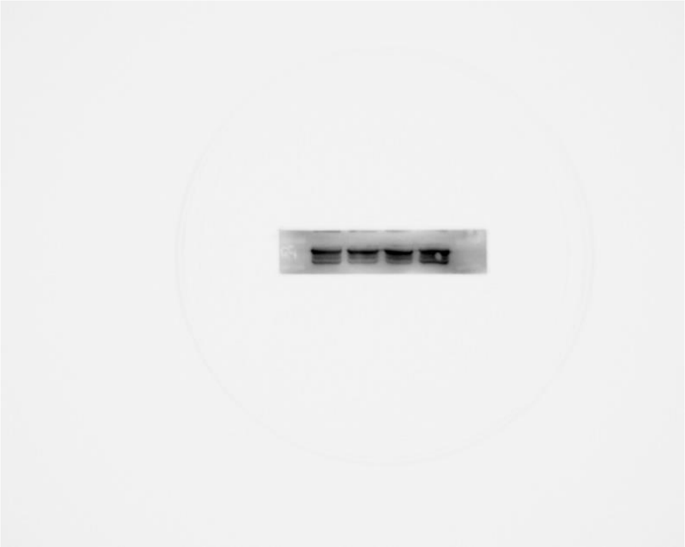

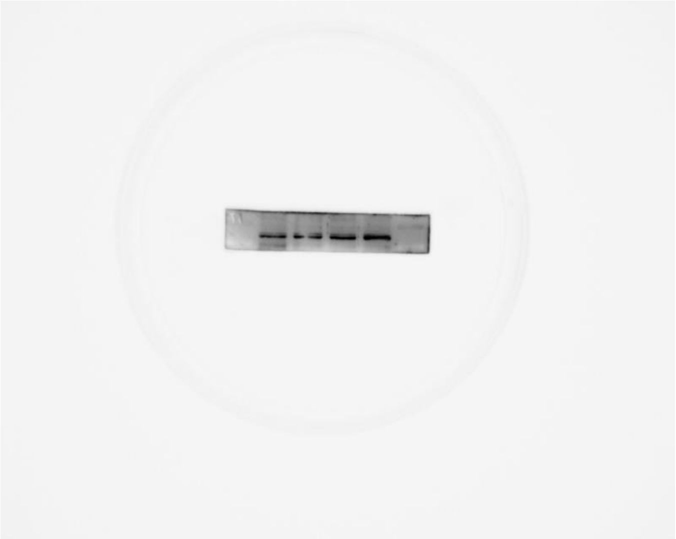

Bcl-2:

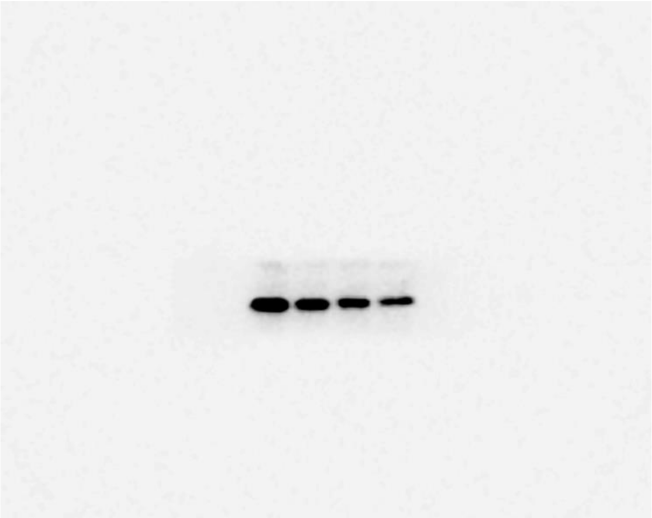

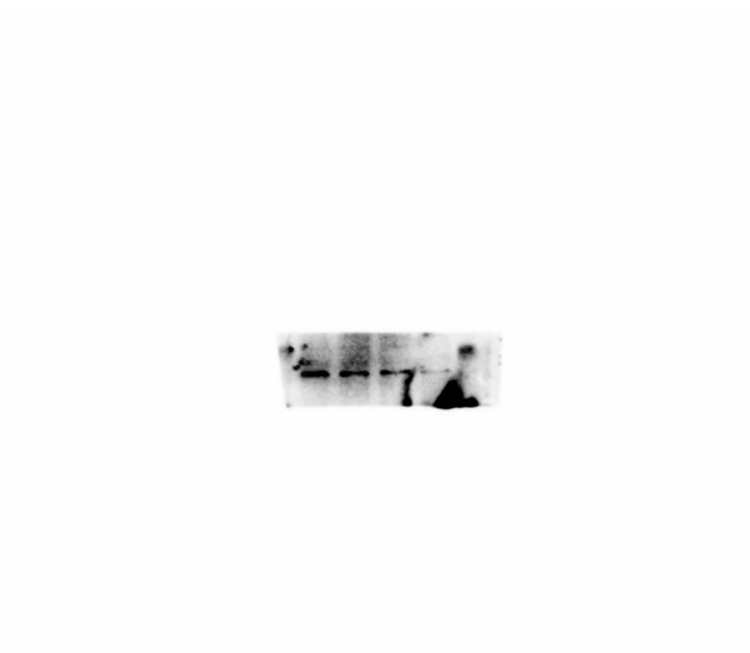

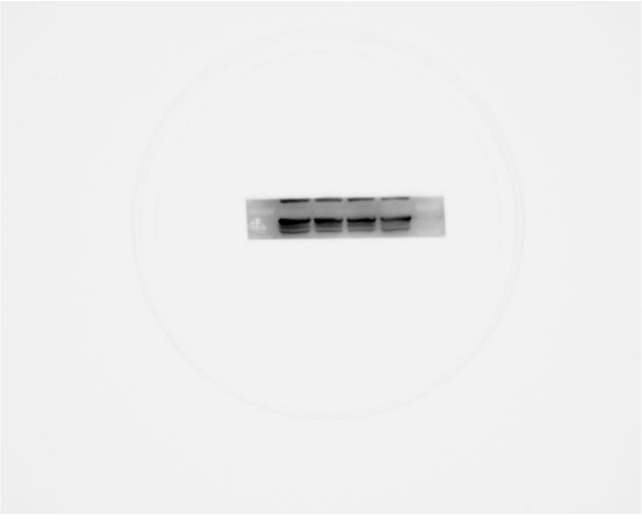

Active-caspase 3:

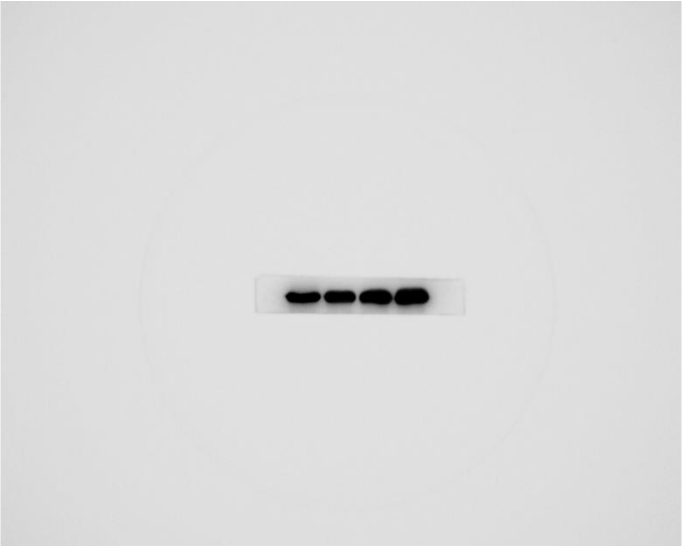

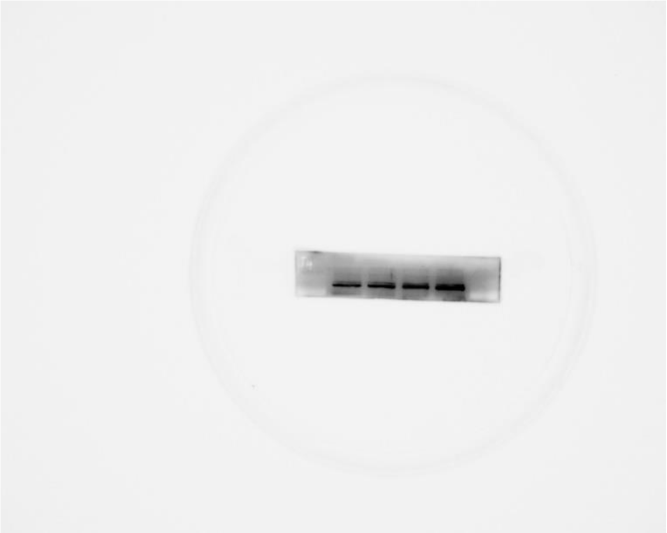

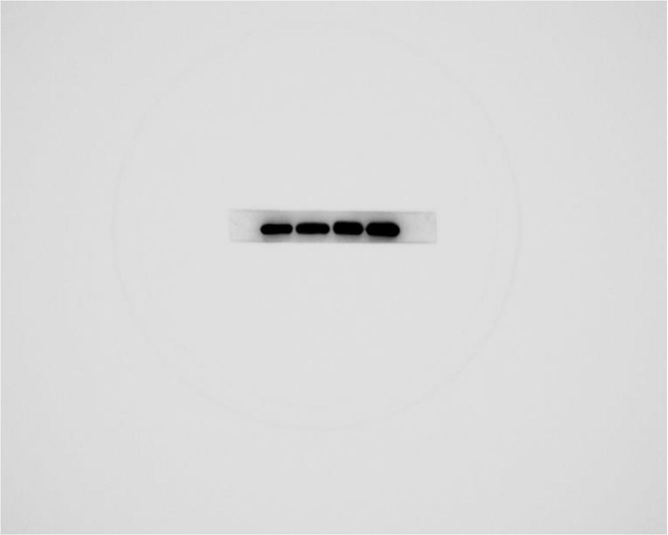

Sirt1:

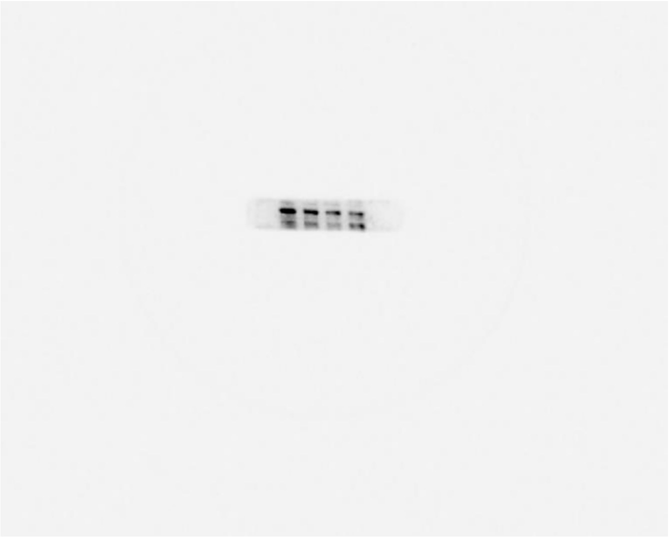

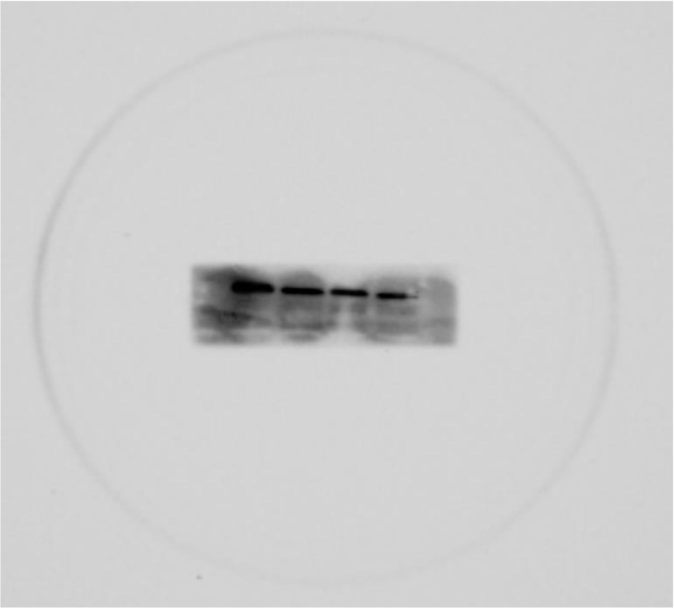

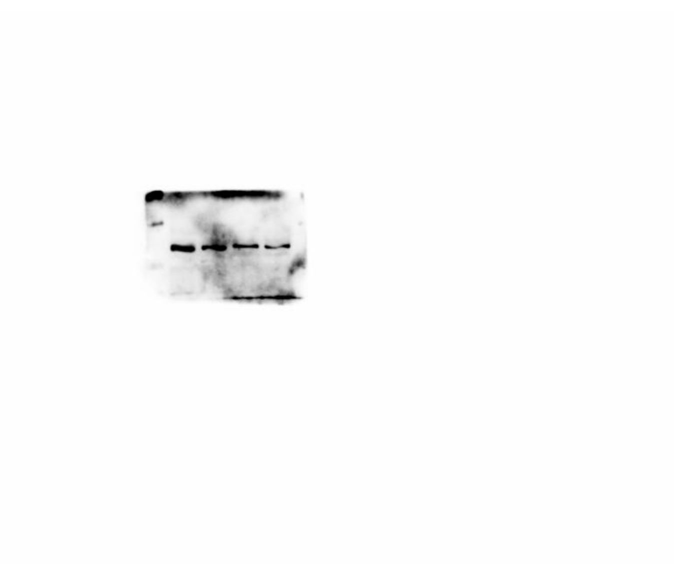

PGC-1α:

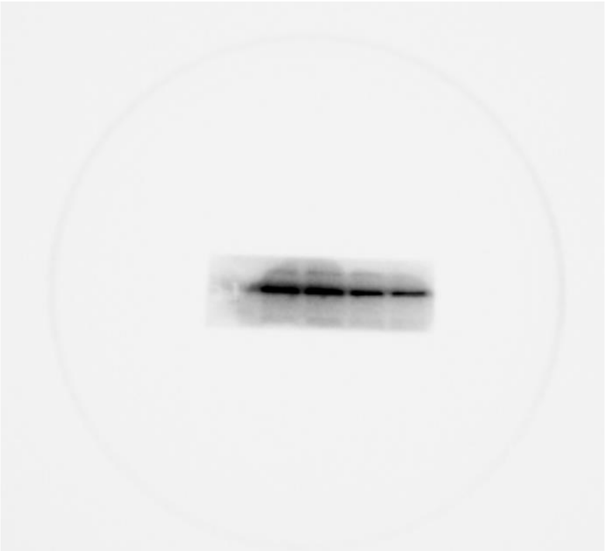

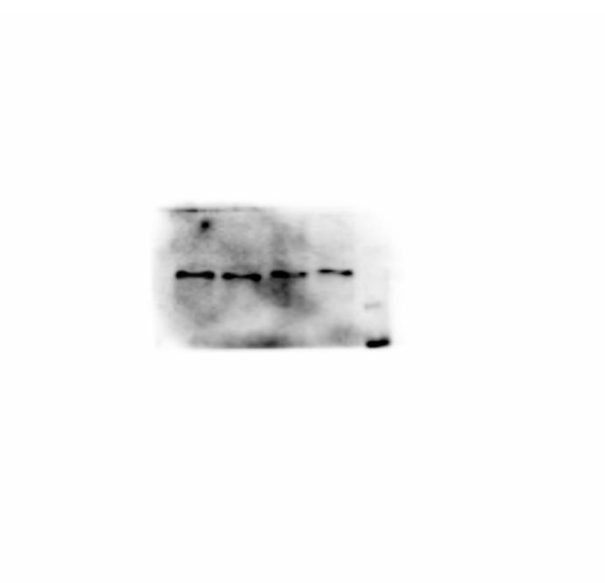

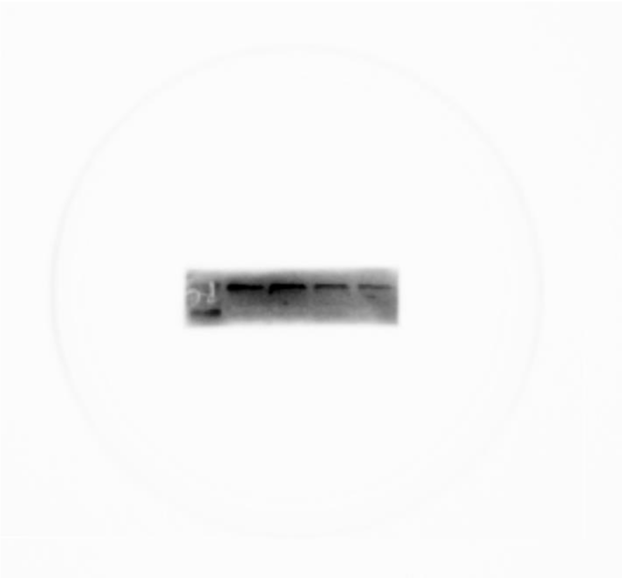

β-actin:

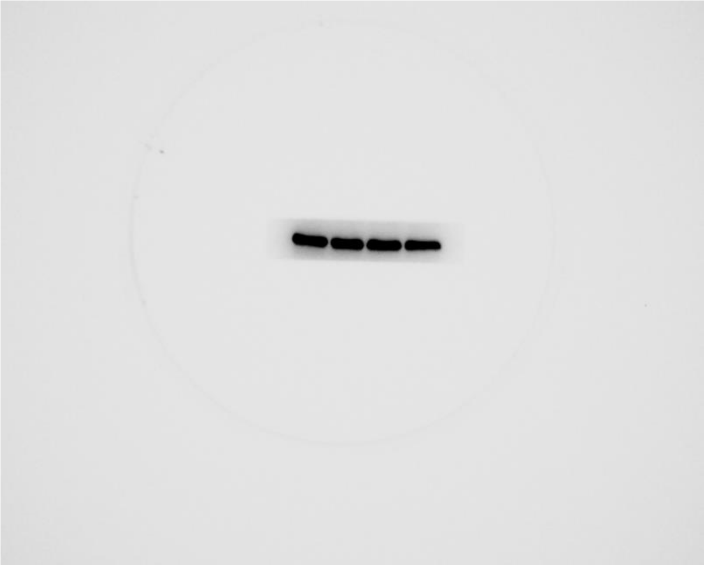

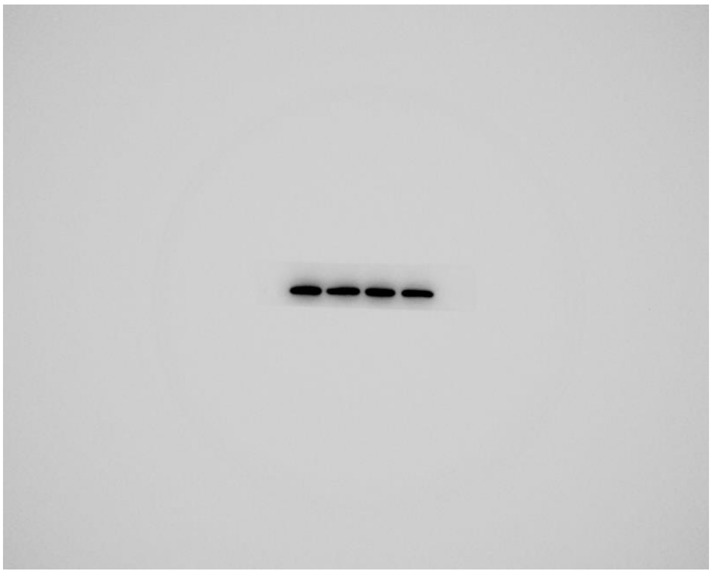

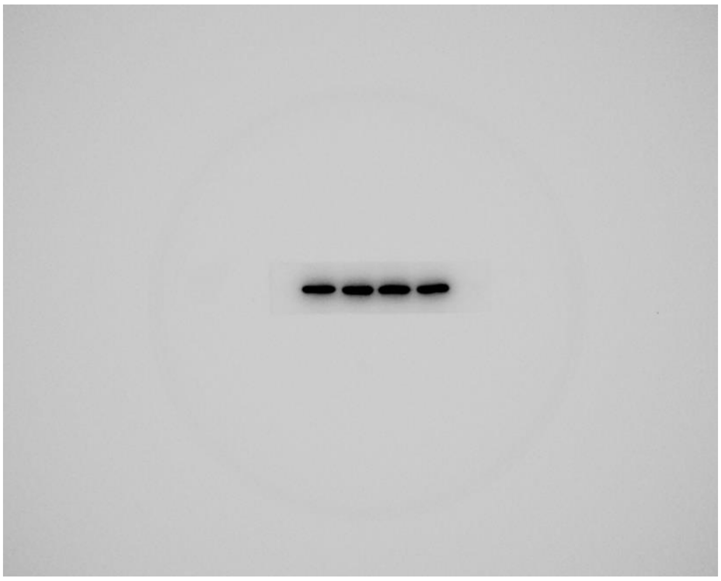

Supplement: Supplementary file 2 [file DataSheet_2.pdf]
